# Second‐trimester transvaginal ultrasound measurement of cervical length for prediction of preterm birth: a blinded prospective multicentre diagnostic accuracy study

**DOI:** 10.1111/1471-0528.16519

**Published:** 2020-10-19

**Authors:** P Kuusela, B Jacobsson, H Hagberg, H Fadl, P Lindgren, J Wesström, U‐B Wennerholm, L Valentin

**Affiliations:** ^1^ Department of Obstetrics and Gynaecology Centre of Perinatal Medicine and Health Institute of Clinical Sciences Sahlgrenska Academy University of Gothenburg Gothenburg Sweden; ^2^ Södra Älvsborg Hospital Borås Sweden; ^3^ Department of Obstetrics Region Vastra Gotaland Sahlgrenska University Hospital Gothenburg Sweden; ^4^ Department of Obstetrics and Gynaecology Faculty of Medicine and Health Örebro University Örebro Sweden; ^5^ Department of Clinical Science, Intervention and Technology Karolinska Institute Stockholm Sweden; ^6^ Centre for Fetal Medicine Karolinska University Hospital Stockholm Sweden; ^7^ Centre for Clinical Research Dalarna Falun Hospital Falun Sweden; ^8^ Department of Obstetrics and Gynaecology Skåne University Hospital Malmö Sweden; ^9^ Department of Medical Sciences Malmö Lund University Lund Sweden

**Keywords:** Cervical length measurement, diagnostic screening programmes, pregnancy, preterm birth, second trimester

## Abstract

**Objective:**

To estimate the diagnostic performance of sonographic cervical length for the prediction of preterm birth (PTB).

**Design:**

Prospective observational multicentre study.

**Setting:**

Seven Swedish ultrasound centres.

**Sample:**

A cohort of 11 456 asymptomatic women with a singleton pregnancy.

**Methods:**

Cervical length was measured with transvaginal ultrasound at 18–20 weeks of gestation (C×1) and at 21–23 weeks of gestation (C×2, optional). Staff and participants were blinded to results.

**Main outcome measures:**

Area under receiver operating characteristic curve (AUC), sensitivity, specificity, positive and negative predictive values (PPV and NPV), positive and negative likelihood ratios (LR+ and LR−), number of false‐positive results per true‐positive result (FP/TP), number needed to screen to detect one PTB (NNS) and prevalence of ‘short’ cervix.

**Results:**

Spontaneous PTB (sPTB) at <33 weeks of gestation occurred in 56/11 072 (0.5%) women in the C×1 population (89% white) and in 26/6288 (0.4%) in the C×2 population (92% white). The discriminative ability of shortest endocervical length was better the earlier the sPTB occurred and was better at C×2 than at C×1 (AUC to predict sPTB at <33 weeks of gestation 0.76 versus 0.65, difference in AUC 0.11, 95% CI 0.01–0.23). At C×2, the shortest endocervical length of ≤25 mm (prevalence 4.4%) predicted sPTB at <33 weeks of gestation with sensitivity 38.5% (10/26), specificity 95.8% (5998/6262), PPV 3.6% (10/274), NPV 99.7% (5988/6014), LR+ 9.1, LR− 0.64, FP/TP 26 and NNS 629.

**Conclusions:**

Second‐trimester sonographic cervical length can identify women at high risk of sPTB. In a population of mainly white women with a low prevalence of sPTB its diagnostic performance is at best moderate.

**Tweetable abstract:**

Cervical length screening to predict preterm birth in a white low‐risk population has moderate performance.

## Tweetable abstract

Cervical length screening to predict preterm birth in a white low‐risk population has moderate performance.

## Introduction

Neonatal morbidity and mortality are inversely proportional to gestational age at birth.^1^ Birth at <33 weeks of gestation is associated with reduced survival, serious medical disabilities and poorer socio‐economic outcomes in adulthood.^2^, ^3^, ^4^, ^5^ In 2016, 0.9% of singleton births in Sweden occurred at <33 weeks of gestation (www.socialstyrelsen.se/sok/?q=graviditeter%2C+f%C3%B6rlossningar+och+nyf%C3%B6dda+barn).

A ‘short cervix’ measured with transvaginal ultrasound in the second trimester increases the likelihood of spontaneous preterm birth (sPTB) in singleton pregnancies.^6^ Treating women with a singleton pregnancy and short cervix in the second trimester with vaginal progesterone may result in a 30% reduction in the number of sPTBs at <33 weeks of gestation.^7^ A short cervix is often defined as ≤25 mm, but other cut‐off values have been suggested.^6^, ^7^, ^8^, ^9^, ^10^ Universal screening of singleton pregnancies with transvaginal ultrasound measurement of cervical length added to the routine second‐trimester ultrasound examination has been advocated.^8^

To estimate the effectiveness of universal screening, one must first determine how well cervical length measured by ultrasound in the second trimester can discriminate between asymptomatic women with a singleton pregnancy, in the general pregnant population, who will and will not experience sPTB. Our systematic literature search yielded five blinded studies estimating this ability.^6^, ^11^, ^12^, ^13^, ^14^ The disparate results of these studies are probably explained by differences in measurement technique, gestational age at cervical length measurement and the characteristics of the study populations (race, ethnicity, socio‐economic status). On the basis of the published data we found it impossible to estimate the ability of cervical length to correctly predict PTB in a population of mainly white women with a low prevalence of PTB. This information is needed before implementing universal cervical screening in such a population.

The aim of this study is to estimate the diagnostic performance of second‐trimester sonographic cervical length for the prediction of PTB in asymptomatic women with a singleton pregnancy.

## Methods

### Study design and participants

This is a prospective blinded multicentre diagnostic accuracy study conducted at six university hospitals and one regional hospital in Sweden. Consecutive women attending a routine second‐trimester ultrasound examination were recruited between May 2014 and June 2017. Women ≥18 years of age with a live singleton pregnancy between 18^+0^ and 20^+6^ weeks of gestation were invited to participate. Information leaflets were available in eight languages. Written informed consent was obtained from all participants. Gestational age was estimated on the basis of ultrasound measurement of the fetal biparietal diameter,^15^, ^16^ or on the day of embryo transfer in the case of in vitro fertilisation, as recommended in the Swedish guidelines (www.sfog.se/media/336451/fetometri.pdf). Exclusion criteria were: fetal malformations detected at the scan; ruptured membranes detected at the scan; bleeding or other clinical signs of miscarriage; current use of progesterone; cerclage in situ; difficulties with understanding written or oral study information; medical termination of pregnancy after registration in the study; and missing information about pregnancy outcome. A woman could participate with only one pregnancy in the study.

The study protocol included two measurements of cervical length: one between 18^+0^ and 20^+6^ weeks of gestation (C×1), performed on the day of the routine scan, and another between 21^+0^ and 23^+6^ weeks of gestation (C×2, optional), with at least 14 days between the two measurements. Women who declined cervical length measurement but fulfilled our inclusion criteria and allowed us to collect information on their background characteristics and pregnancy outcome comprise our ‘no cervix measurement’ control group. To estimate selection bias, background data and pregnancy outcome of our study population were compared with those of the background population, comprising women of ≥ 18 years of age with a singleton pregnancy who gave birth in Sweden during the study period (with the first singleton delivery occurring during the period from the first to the last delivery in the study population).

### Procedures

In Sweden, routine second‐trimester ultrasound examinations are scheduled at 18 weeks of gestation. They are performed by specially trained midwife sonographers, certified by the Swedish Society of Obstetricians and Gynaecologists to perform routine fetal ultrasound examinations after standardised theoretical education and practical training, and after having passed a theoretical and practical test. The cervical length measurements were performed by 25 midwife sonographers who had also been certified to perform cervical length measurements.^17^ After certification, quality controls were performed four times a year. A midwife sonographer that failed three subsequent quality checks was no longer allowed to examine study participants.

**Figure 1:**
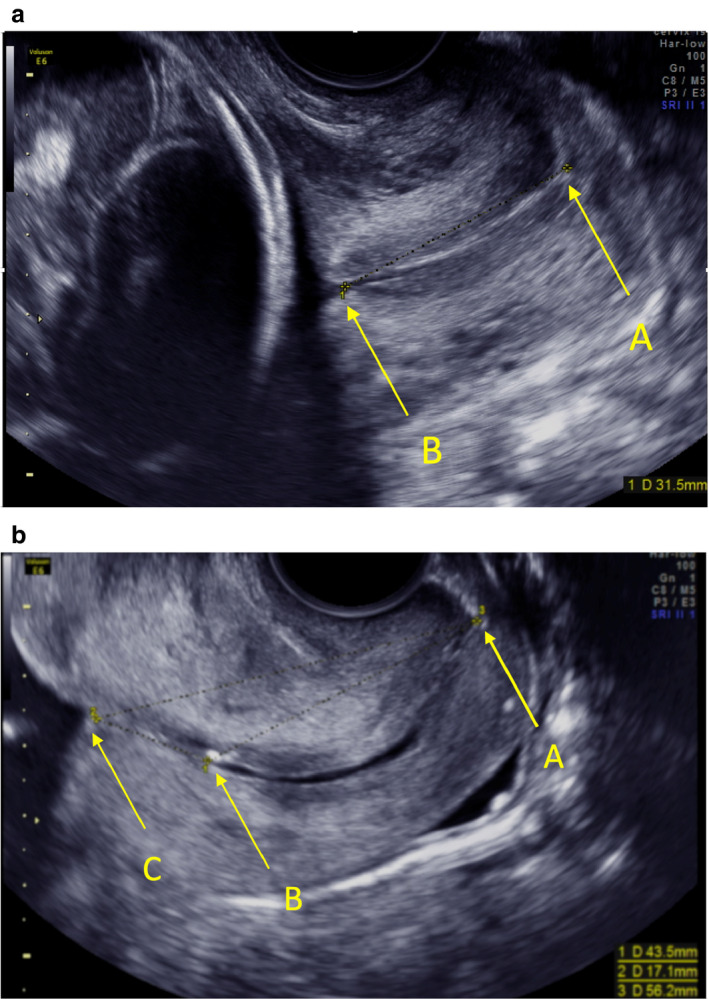
Measurement of cervical length when isthmus is absent (a) or present (b). Isthmus is the lowest part of the uterine corpus that develops into the lower uterine segment as pregnancy progresses. The letter ‘A’ denotes the external os. ‘B’ denotes the internal os. ‘C’ (which we call the ‘virtual inner os’) is the innermost end of the juxtaposed anterior and posterior isthmus. Measurements were taken as a straight line from A to B (endocervical length), from B to C (isthmus length) and from A to C. In the main text, we present results for the shortest of three measurements of endocervical length (A–B). In supporting tables we also present results for mean and maximum A–B, and for minimum, mean and maximum A–C and A–B + B–C.

The study participants were examined in the lithotomy position in a gynaecological chair with an empty urinary bladder. The transvaginal probe was introduced into the vagina and a sagittal view of the cervix was obtained. Efforts were made to obtain images fulfilling five quality criteria: (i) the cervix occupies at least 75% of the screen; (ii) the anterior and posterior lip of the cervix are of equal thickness; (iii) the full length of the endocervical canal is clearly seen; (iv) the inner and outer cervical os are clearly seen, as well as the virtual inner os if the isthmus is present (the isthmus is the lowest part of the uterine corpus that develops into the lower uterine segment as pregnancy progresses); and (v) callipers are positioned correctly at the internal and external os, and at the virtual inner os if the isthmus is present. If the isthmus was present, three distances were measured: endocervical length, distance A–B; isthmus length, distance B–C; and distance A–C (Figure 1). Funnelling of the cervix was not recorded and fundal or suprapubic pressure was not applied. Three measurements of each distance were taken during a period of at least 3 min, with each measurement being taken on a new image. All distances were recorded in millimetres without decimals. The measurement results were registered in a web‐based electronic case record form (MedSciNet AB, Stockholm, Sweden), together with anamnestic information obtained from the women. If cervical length could not be measured because of the woman’s discomfort or poor image quality, the result was denoted ‘not measurable’ and the reason recorded. It was obligatory to store electronic still images of all measurements. All ultrasound examinations were carried out using a GE Healthcare Voluson E8 Expert or E6 ultrasound system, with a 5–9 MHz vaginal transducer (GE Corporate, Fairfield, CT, USA). Both medical staff and participants were blinded to the cervical length results. They were only disclosed if the amniotic sac bulged into the vagina, indicating imminent miscarriage.

We obtained information on background data and pregnancy outcome from the Swedish Pregnancy Register (www.graviditetsregistret.se),^18^ which covered 93% of deliveries in Sweden in 2017. To obtain information on redeemed prescription of vaginal progesterone in the current pregnancy, cervical conisation and cerclage and history of PTB, we used information from three registers hosted by the Swedish National Board of Health and Welfare (www.socialstyrelsen.se): The Swedish Prescribed Drug Register; the Swedish National Patient Register (validated by Ludvigsson et al.^19^); and The Swedish Medical Birth Register (validated by Cnattingius et al. 1990^20^; https://www.socialstyrelsen.se/globalassets/sharepoint-dokument/artikelkatalog/ovrigt/2003-112-3_20031123.pdf). Information on the participants’ own perceptions of their ethnicity was obtained from the electronic case record form. If delivery data for the study populations was missing in the Swedish Pregnancy Register, we searched information in the medical records. If no information was found, the participant was contacted by mail or telephone.

### Reference standard

Our reference standard is PTB, i.e. birth before 37^+0^ weeks of gestation. We define sPTB as birth after spontaneous start of labour (International Classification of Diseases, tenth revision, ICD‐10, code O60.1) or after preterm prelabour rupture of the membranes (ICD‐10 code O42), regardless of whether labour was induced. The medical records of all preterm births in the C×1 and C×2 populations were scrutinised for validation of the sPTB diagnosis, with the assessor being blinded to the results of the cervical length measurements.

Our primary outcome was PTB between 22^+0^ and 32^+6^ weeks of gestation, including stillbirths. We chose birth at <33 weeks of gestation as our primary outcome, because birth at <33 weeks of gestation is associated with a high risk of short‐term and long‐term complications.^2^, ^3^, ^4^, ^5^ Our secondary outcomes were sPTB at <28, <29, <30, <31, <32, <33, <34, <35, <36 or <37 completed weeks of gestation, including both stillbirths and late miscarriages, i.e. miscarriages occurring between 18^+0^ and 21^+6^ weeks of gestation.

Pregnant women were not involved in developing the study design, in the interpretation of the data or in the writing up of the results. There is no core outcome set for diagnostic accuracy studies.

### Statistical analysis

We followed a pre‐specified statistical analysis plan. We present descriptive data as mean (SD), median, interquartile range (IQR), minimum and maximum for continuous variables, and as numbers and percentages for categorical variables.

We estimated the effect of cervical length on sPTB (odds ratio per mm increase in cervical length) by calculating odds ratios with 95% confidence intervals (95% CIs) using logistic regression. The effect of cervical length on sPTB was also studied per participating centre. To test whether the effect of cervical length on sPTB was similar across centres, we performed an interaction analysis using logistic regression with sPTB as a dependent variable and with cervical length, centre and centre*cervical length as independent variables.

We use Kaplan–Meier plots to illustrate the proportion of women still pregnant at different gestational ages for different cervical lengths at C×1 and C×2, indicated PTBs being censored.

We describe the ability of the cervical measurements and changes in the measurements between C×1 and C×2 (in mm as well as in percentage of the C×1 measurement) to discriminate between women who deliver before or after a defined gestational week as area under the receiver operating characteristic (ROC) curve (AUC),^21^, ^22^ sensitivity, specificity, positive and negative predictive values (PPV and NPV), positive and negative likelihood ratios (LR+ and LR−), number of false‐positive test results per one true‐positive test result (FP/TP) and number needed to screen (NNS) to correctly identify one PTB. If the lower limit of the 95% CI of the AUC is >0.5, we consider the measurement to have discriminative potential. We consider the measurement with the largest AUC to have the greatest discriminative ability. To calculate the 95% CI of difference in AUC we use bootstrapping (1000 samples). We also use the ROC curves to identify the measurement cut‐off to predict PTB that yields the highest proportion of correctly classified cases (Youden’s index).^21^ We call this cut‐off the ‘best cut‐off’. In addition, we estimate the discriminative ability of cervical lengths of ≤15, ≤20, ≤25 and ≤30 mm, as others have suggested the use of these cut‐offs.^6^, ^7^, ^8^, ^9^, ^10^, ^23^

We estimated the sample size in the following manner. We needed to strike a balance between a sample size that was possible to achieve and our wish to get precise estimates of sensitivity with regard to PTB at <33 weeks of gestation. We looked at the 95% CI around point estimates for sensitivities between 10 and 90%, and found it to be acceptable with 100 individuals in the denominator. We expected 0.9% of all deliveries to occur at <33^+0^ weeks of gestation (www.socialstyrelsen.se/sok/?q=graviditeter%2C+f%C3%B6rlossningar+och+nyf%C3%B6dda+barn). This means that to find 100 births at <33 weeks of gestation we needed to perform cervical length measurements in 11 000 women.

Statistical calculations were performed using sas 9.4 (SAS‐Institute, Cary, NC, USA).

## Results

**Figure 2:**
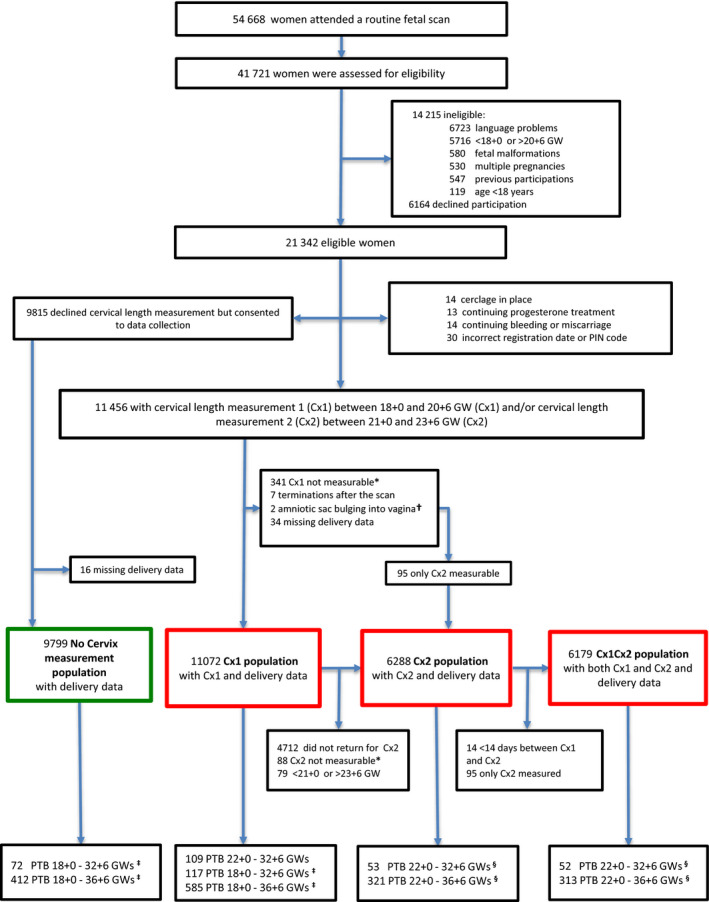
Flow chart showing the study populations. *In some women, their discomfort made it impossible to measure the cervix. †Excluded after C×1 measurement. One woman got a cerclage and gave birth at 33 weeks of gestation, one woman miscarried after 2 days of bed rest. ‡Includes miscarriage between 18^+0^ and 21^+6^ weeks of gestation (in the study groups we include miscarriages that occurred after the cervix measurement). §There were no miscarriages between 21^+0^ and 21^+6^ weeks of gestation, so preterm birth does not include late miscarriage here. Abbreviations: GW, gestational weeks; PIN, personal identity number; PTB, preterm birth.

Figure 2 shows patient flow. Our study populations comprise 11 072 women with delivery data and results for C×1 (C×1 population), 6288 women with delivery data and results for C×2 (C×2 population) and 6179 women with delivery data and results for both C×1 and C×2, with at least 14 days between the two measurements (C×1 C×2 population). Our ‘no cervix measurement’ population includes 9799 women with delivery data, our background population includes 347 479 women. Tables S1 and S2 show background and outcome data for our study and control populations. In our three study populations (C×1, C×2, C×1 C×2), the proportions of women born outside Europe were 9.9, 7.3 and 7.3%, respectively, versus 9.5% in our ‘no cervix measurement’ population and 18.2% in our background population. The proportions of women with previous cervical conisation were 5.9, 6.4 and 6.4%, respectively, versus 4.2 and 4.3%, respectively. The proportions of women with previous sPTB of a singleton pregnancy were 3.4, 3.5 and 3.6%, respectively, versus 2.1 and 2.3%, respectively. In our study populations, 3.6, 3.6 and 3.7% of the women delivered spontaneously at <37 weeks of gestation, versus 3.0 and 3.2% in our control populations. After inclusion in the study, 18/11 072 (0.2%) women in the C×1 population and 9/6288 (0.1%) women in the C×2 population were treated with progesterone, and one woman had a cerclage inserted (Table S2).

The median gestational age at C×1 was 19^+0^ weeks of gestation (IQR 18^+3^–19^+3^ weeks of gestation; min. 18^+0^ and max. 20^+6^ weeks of gestation) and the median gestational age at C×2 was 23^+0^ weeks of gestation (IQR 22^+4^–23^+3^ weeks of gestation; min. 21^+0^ and max. 23^+6^ weeks of gestation). The number of days between the C×1 and C×2 measurement was a median of 28 days (IQR 24–31 days; min. 14 and max. 41 days). The isthmus was present in 23.3% (2566/11 072) of the women at C×1 and in 8.9% (557/6288) of the women at C×2.

Isthmus length had no or very poor ability to discriminate between women who did and did not deliver preterm or spontaneously preterm at any defined gestational age between <28 and <37 weeks of gestation. The discriminative ability of the other measurements was similar, and this was also true in women with the isthmus present (Tables [Link], [Link], [Link]). Below we present results for shortest endocervical length (distance A–B), which is a commonly used measurement to identify women at increased risk of PTB.^6^, ^11^, ^12^, ^13^, ^14^

The median shortest endocervical length at C×1 was 36.0 mm (IQR 32.0–40.0 mm; min. 3.0 and max. 60.0 mm) and the median shortest endocervical length at C×2 was 36.0 mm (IQR 32.0–40.0 mm; min. 4.0 and max. 60.0 mm). At C×1, 67/11 072 (0.6%) women had a cervical length of ≤20 mm and 441/11 072 (4.0%) had a cervical length of ≤25 mm. At C×2, 71/6288 (1.1%) women had a cervical length of ≤20 mm and 274 (4.4%) had a cervical length of ≤25 mm (Table S6).

For sPTB at <33 weeks of gestation, the odds ratio for shortest endocervical length (per mm increase) at C×1 was 0.87 (95% CI 0.84–0.90) and at C×2 was 0.83 (95% CI 0.79–0.87). For sPTB at <37 weeks of gestation, the corresponding odds ratios were 0.94 (95% CI 0.92–0.95) and 0.92 (95% CI 0.90–0.94). The odds ratios were similar across centres, without significant interaction effects. For sPTB at <33 weeks of gestation the odds ratios at C×1 ranged from 0.82 to 0.92 in the different centres, and at C×2 they ranged from 0.67 to 0.88. For sPTB at <37 weeks of gestation they ranged from 0.90 to 0.97 at C×1 and from 0.87 to 0.94 at C×2. The Kaplan–Meier plots (Figures S1 and S2) illustrate that the shorter the cervix at C×1 and C×2, the shorter the time to sPTB.

The ability of shortest endocervical length at C×1 and C×2 to correctly predict PTB between 22^+0^ and 32^+6^ weeks of gestation (primary outcome) was poor (AUC 0.57, 95% CI 0.51–0.63 for C×1; AUC 0.59, 95% CI 0.50–0.68 for C×2). The ability to correctly predict sPTB (including late miscarriage) was better but decreased with advancing gestational age at birth (Tables 1 and 2). The discriminative ability at C×2 was superior to that at C×1. This was confirmed in the C×1 C×2 population (AUC 0.76 versus 0.65, difference in AUC 0.11, 95% CI 0.01–0.23), in which we also found that a change in cervical length between C×1 and C×2 had discriminative ability similar to or poorer than that of a single measurement (Table S7). The discriminative ability of shortest endocervical length was poorer in women with than without isthmus (AUC 0.57 versus 0.74 for prediction of sPTB at <33 weeks of gestation at C×1; Table S5).

**Table 1:** Discriminative ability of shortest endocervical length (distance A–B) measured at 18^+0^–20^+6^ weeks of gestation (C×1, *n* = 11 072) with regard to predicting spontaneous preterm birth, including late spontaneous miscarriage

sPTB	No. sPTB (%)	AUC [95% CI]	Cervical length												
			0–15 mm (*n* = 15; 0.14%)			0–20 mm (*n* = 67; 0.6%)			0–25 mm (*n* = 441; 4.0%)			Best cut‐off*			
			Sensitivity [95% CI]	Specificity [95% CI]	FP/TP; NNS	Sensitivity [95% CI]	Specificity [95% CI]	FP/TP; NNS	Sensitivity [95% CI]	Specificity [95% CI]	FP/TP; NNS	Sensitivity [95% CI]	Specificity [95% CI]	FP/TP; NNS	mm
<28 GW	22 (0.20%)	0.83 [0.74–0.92]	3/22 (13.6%] [2.9–34.9]	11 038/11 050 (99.9%) [99.8–99.9]	4; 3691	4/22 (18.2) [5.2–40.3]	10 987/11 050 (99.4%) [99.3–99.6]	16; 2768	9/22 (40.9%) [20.7–63.7]	10 618/11 050 (96.1%) [95.7–96.4]	48; 1230	18/22 (81.8%) [59.7–94.8]	7685/11 050 (69.5%) [68.7–70.4]	187; 615	32 (*n* = 3383; 30.6%)
<29 GW	24 (0.22%)	0.84 [0.76–0.92]	3/24 (12.5%) [2.7–32.4]	11 036/11 048 (99.9%) [99.8–99.9]	4; 3691	5/24 (20.8%) [7.1–42.2]	10 986/11 048 (99.4%) [99.3–99.6]	12; 2214	10/24 (41.7%) [22.1–63.4]	10 617/11 048 (96.1%) [95.7–96.5]	43; 1107	20/24 (83.3%) [62.6–95.3]	7685/11 048 (69.6%) [68.7–70.4]	168; 554	32 (*n* = 3383; 30.6%)
<30 GW	34 (0.31%)	0.77 [0.68–0.87]	3/34 (8.8%) [1.9–23.7]	11 026/11 038 (99.9%) [99.8–99.9]	4; 3691	5/34 (14.7%) [5.0–31.1]	10 976/11 038 (99.4%) [99.3–99.6]	12; 2214	12/34 (35.3%) [19.8–53.5]	10 609/11 038 (96.1%) [95.7–96.5]	36; 923	25/34 (73.5%) [55.6–87.1]	7680/11 038 (69.6%) [68.7–70.4]	134; 443	32 (*n* = 3383; 30.6%)
<31 GW	40 (0.36%)	0.76 [0.67–0.85]	3/40 (7.5%) [1.6–20.4]	11 020/11 032 (99.9%) [99.8–99.9]	4; 3691	6/40 (15.0%) [5.7–29.8]	10 971/11 032 (99.4%) [99.3–99.6]	10; 1845	13/40 (32.5%) [18.6–49.1]	10 604/11 032 (96.1%) [95.7–96.5]	33; 852	29/40 (72.5%) [56.1–85.4]	7678/11 032 (69.6%) [68.7–70.5]	116; 382	32 (*n* = 3383; 30.6%)
<32 GW	46 (0.42%)	0.71 [0.62–0.80]	3/46 (6.5%) [1.4–17.9]	11 014/11 026 (99.9%) [99.8–99.9]	4; 3691	6/46 (13.0%) [4.9–26.3]	10 965/11 026 (99.4%) [99.3–99.6]	10; 1845	14/46 (30.4%) [17.7–45.8]	10 599/11 026 (96.1%) [95.8–96.5]	31; 791	23/46 (50.0%) [34.9–65.1]	9411/11 026 (85.4%) [84.7–86.0]	70; 481	29 (*n* = 1638; 14.8%)
<33 GW	63 (0.57%)	0.68 [0.60–0.76]	3/63 (4.8%) [1.0–13.3]	10 997/11 009 (99.9%) [99.8–99.9]	4; 3691	7/63 (11.1%) [4.6–21.6]	10 949/11 009 (99.5%) [99.3–99.6]	9; 1582	17/63 (27.0%) [16.6–39.7]	10 585/11 009 (96.1%) [95.8–96.5]	25; 651	27/63 (42.9%) [30.5–56.0]	9398/11 009 (85.4%) [84.7–86.0]	60; 410	29 (*n* = 1638; 14.8%)
<33 GW**	56 (0.51%**)	0.66 [0.58–0.74]	1/56 (1.8%) [0.1–9.6]	10 996/11 008 (99.9%) [99.8–99.9]	12; 11 064	5/56 (8.9%) [8.9–19.6]	10 948/11 008 (99.5%) [99.3–99.6]	12; 2213	13/56 (23.2%) [13.0–36.4]	10 584/11 008 (96.1%) [95.8–96.5]	33; 851	38/56 (67.9%) [54.0–79.7]	6364/11 008 (57.8%) [56.9–58.7]	122; 291	34 (*n* = 4682; 42.3%)
<34 GW	94 (0.85%)	0.65 [0.59–0.72]	3/94 (3.2%) [0.7–9.0]	10 966/10 978 (99.9%) [99.8–99.9]	4; 3691	7/94 (7.4%) [3.1–14.7]	10 918/10 978 (99.5%) [99.3–99.6]	9; 1582	20/94 (21.3%) [13.5–30.9]	10 557/10 978 (96.2%) [95.8–96.5]	21; 554	35/94 (37.2%) [27.5–47.8]	9375/10 978 (85.4%) [84.7–86.1]	46; 316	29 (*n* = 1638; 14.8%)
<35 GW	143 (1.29%)	0.62 [0.57–0.67]	4/143 (2.8%) [0.8–7.0]	10 918/10 929 (99.9%) [99.8–100.0]	3; 2768	8/143 (5.6%) [2.5–10.7]	10 870/10 929 (99.5%) [99.3–99.6]	7; 1384	22/143 (15.4%) [9.9–22.4]	10 510/10 929 (96.2%) [95.8–96.5]	19; 503	69/143 (48.3%) [39.8–56.8]	7615/10 929 (69.7%] [68.8–70.5]	48; 161	32 (*n* = 3383; 30.6%)
<36 GW	226 (2.04%)	0.61 [0.57–0.65]	4/226 (1.8%) [0.5–4.5]	10 835/10 846 (99.9%) [99.8–100.0]	3; 2768	9/226 (4.0%) [1.8–7.4]	10 788/10 846 (99.5%) [99.3–99.6]	6; 1230	30/226 (13.3%) [9.1–18.4]	10 435/10 846 (96.2%) [95.8–96.6]	14; 369	118/226 (52.2%) [45.5–58.9]	6951/10 846 (64.1%) [63.2–65.0]	33; 94	33 (*n* = 4013–36.2%)
<37 GW	417 (3.77%)	0.60 [0.57–0.63]	5/417 (1.2%) [0.4–2.8]	10 645/10 655 (99.9%) [99.8–100.0]	2; 2214	13/417 (3.1%) [1.7–5.3]	10 601/10 655 (99.5%) [99.3–99.6]	4; 852	38/417 (9.1%) [6.5–12.3]	10 252/10 655 (96.2%) [95.8–96.6]	11; 291	207/417 (49.6%) [44.7–54.6]	6849/10 655 (64.3%) [63.4–65.2]	18; 54	33 (*n* = 4013; 36.2%)

Positive and negative predictive values and positive and negative likelihood ratios are shown in Table S8.

GW, gestational weeks; sPTB, spontaneous preterm birth including miscarriage; AUC, area under receiver operating characteristic curve; CI, confidence interval; FP, false positive; TP, true positive; NNS, number needed to screen, i.e. number of women needed to screen to detect one true positive test result.

* Best cut‐off is the cut‐off associated with the largest number of correctly classified cases, calculated using Youden’s index.^21^

** Late miscarriage excluded, with *n* = 11 064 as denominator.

**Table 2:** Discriminative ability of shortest endocervical length (distance A‐B) measured at 21 + 0 to 23 + 6 gestational weeks (C×2, *n* = 6288) with regard to predicting spontaneous preterm birth

sPTB	No. sPTB (%)	AUC [95% CI]	Cervical length												
			0–15 mm (*n* = 14; 0.22%)			0–20 mm (*n* = 71; 1.1%)			0–25 mm (*n* = 274; 4.4%)			Best cut‐off*			
			Sensitivity [95% CI]	Specificity [95% CI]	FP/TP; NNS	Sensitivity [95% CI]	Specificity [95% CI]	FP/TP; NNS	Sensitivity [95% CI]	Specificity [95% CI]	FP/TP; NNS	Sensitivity [95% CI]	Specificity [95% CI]	FP/TP; NNS	mm
<28 GW	3 (0.05)	0.96 [0.92–1.00]	1/3 (33.3%) [0.8–90.6]	6272/6285 (99.8%) [99.7–99.9]	13; 6288	1/3 (33.3%) [0.8–90.6]	6215/6285 (98.9%) [98.6–99.1]	70; 6288	1/3 (33.3%) [0.8–90.6]	6012/6285 (95.7%) [95.1–96.2]	273; 6288	3/3 (100%) [29.2–100]	5778/6285 (91.9%) [91.2–92.6]	169; 2096	27 (*n* = 510; 8.1%)
<29 GW	5 (0.08)	0.98 [0.95–1.00]	3/5 (60.0%) [14.7–94.7]	6272/6283 (99.8%) [99.7–99.9]	4; 2096	3/5 (60.0%) [14.7–94.7]	6215/6283 (98.9%) [98.6–99.2]	23; 2096	3/5 (60.0%) [14.7–94.7]	6012/6283 (95.7%) [95.2–96.2]	90; 2096	5/5 (100%) [47.8–100]	5778/6283 (92.0%) [91.3–92.6]	101; 1258	27 (*n* = 510; 8.1%)
<30 GW	10 (0.16)	0.86 [0.69–1.00]	5/10 (50.0%) [18.7–81.3]	6269/6278 (99.9%) [99.7–99.9]	2; 1257	6/10 (60.0%) [26.2–87.8]	6213/6278 (99.0%) [98.7–99.2]	11; 1048	6/10 (60.0%) [26.3–87.8]	6010/6278 (95.7%) [95.2–96.2]	45; 1048	8/10 (80.0%) [44.4–97.5]	5776/6278 (92.0%) [91.3–92.7]	63; 786	27 (*n* = 510; 8.1%)
<31 GW	15 (0.24)	0.85 [0.72–0.99]	6/15 (40.0%) [16.3–67.7]	6265/6273 (99.9%) [99.8–99.9]	1; 1048	8/15 (53.3%) [26.6–78.7]	6210/6273 (99.0%) [98.7–99.2]	8; 786	8/15 (53.3%) [26.6–78.7]	6007/6273 (95.8%) [95.2–96.2]	33; 786	11/15 (73.3%) [44.9–92.2]	5774/6273 (92.0%) [91.4–92.7]	45; 572	27 (*n* = 510; 8.1%)
<32 GW	18 (0.29)	0.81 [0.68–0.93]	6/18 (33.3%) [13.3–59.0]	6262/6270 (99.9%) [99.8–99.9]	1; 1048	8/18 (44.4%) [21.5–69.2]	6207/6270 (99.0%) [98.7–99.2]	8; 786	8/18 (44.4%) [21.5–69.2]	6004/6270 (95.8%) [95.2–96.2]	33; 786	11/18 (61.1%) [35.8–82.7]	5771/6270 (92.0%) [91.3–92.7]	45; 572	27 (*n* = 510; 8.1%)
<33 GW	26 (0.41)	0.76 [0.65–0.87]	6/26 (23.1%) [9.0–43.7]	6254/6262 (99.9%) [99.8–99.9]	1; 1048	9/26 (34.6%) [17.2–55.7]	6200/6262 (99.0%) [98.7–99.2]	7; 699	10/26 (38.5%) [20.3–59.4]	5998/6262 (95.8%) [95.3–96.3]	26; 629	14/26 (53.8%) [33.4–73.4]	5766/6262 (92.1%) [91.4–92.7]	35; 449	27 (*n* = 510; 8.1%)
<34 GW	41 (0.65)	0.71 [0.63–0.80]	6/41 (14.6%) [5.6–29.2]	6239/6247 (99.9%) [99.8–99.9]	1; 1048	10/41 (24.4%) [12.4–40.3]	6186/6247 (99.0%) [98.8–99.3]	6; 629	12/41 (29.3%) [16.1–45.5]	5985/6247 (95.8%) [95.3–96.3]	22; 524	16/41 (39.0%) [24.2–55.5]	5753/6247 (92.1%) [91.4–92.8]	31; 393	27 (*n* = 510; 8.1%)
<35 GW	69 (1.10)	0.71 [0.65–0.77]	7/69 (10.1%) [4.2–19.8]	6212/6219 (99.9%) [99.8–100.0]	1; 898	13/69 (18.8%) [10.4–30.1]	6161/6219 (99.1%) [98.8–99.3]	5; 484	17/69 (24.6%) [15.1–36.5]	5962/6219 (95.9%) [95.3–96.4]	15; 370	53/69 (76.8%) [65.1–86.1]	3346/6219 (53.8%) [52.6–55.1]	54; 119	35 (*n* = 2926; 46.5%)
<36 GW	114 (1.81)	0.67 [0.61–0.72]	7/114 (6.1%) [2.5–12.2]	6167/6174 (99.9%) [99.8–100.0]	1; 898	15/114 (13.2%) [7.6–20.8]	6118/6174 (99.1%) [98.8–99.3]	4; 419	23/114 (20.2%) [13.2–28.7]	5923/6174 (95.9%) [95.4–96.4]	11; 273	81/114 (71.1%) [61.8–79.2]	3329/6174 (53.9%) [52.7–55.2]	35; 78	35 (*n* = 2926; 46.5%)
<37 GW	225 (3.58)	0.63 [0.59–0.67]	7/225 (3.1%) [1.3–6.3]	6056/6063 (99.9%) [99.8–100.0]	1; 898	18/225 (8.0%) [4.8–12.4]	6010/6063 (99.1%) [98.9–99.3]	3; 349	34/225 (15.1%) [10.7–20.5]	5823/6063 (96.0%) [95.5–96.5]	7; 185	144/225 (64.0%) [57.4–70.3]	3281/6063 (54.1%) [52.9–55.4]	19; 44	35 (*n* = 2926; 46.5%)

Positive and negative predictive values and positive and negative likelihood ratios are shown in Table S9.

GW, gestational weeks; sPTB, spontaneous preterm birth; AUC, area under receiver operating characteristic curve; CI, confidence interval; FP, false positive; TP, true positive; NNS, number needed to screen, i.e. number of women needed to screen to detect one true positive test result.

* Best cut‐off is the cut‐off associated with the largest number of correctly classified cases, calculated using Youden’s index.^21^

At C×1, a shortest endocervical length of ≤25 mm (prevalence 4.0%) predicted sPTB at <33 weeks of gestation with sensitivity 27%, specificity 96.1%, PPV 3.9% (17/441), NPV 99.6% (10 585/10 631), LR+ 7.0, LR− 0.76, FP/TP 25 and NNS 651. A shortest endocervical length of ≤29 mm (best cut‐off, prevalence 14.8%) had sensitivity 42.9%, specificity 85.4%, PPV 1.6% (27/1638), NPV 99.6% (9398/9434), LR+ 2.9, LR− 0.67, FP/TP 60 and NNS 410 (Tables 1 and S8). At C×2, a shortest endocervical length of ≤25 mm (prevalence 4.4%) predicted sPTB at <33 weeks of gestation with sensitivity 38.5%, specificity 95.8%, PPV 3.6% (10/274), NPV 99.7% (5988/6014), LR+ 9.1, LR− 0.64, FP/TP 26 and NNS 629. A shortest endocervical length of ≤27 mm (best cut‐off, prevalence 8.1%) had sensitivity 53.8%, specificity 92.1%, PPV 2.7% (14/510), NPV 99.8%% (5766/5578), LR+ 6.8, LR− 0.50, FP/TP 35 and NNS 449 (Tables 2 and S9). Results for the 30‐mm cervical length cut‐off are presented in Tables S10 (C×1) and S11 (C×2).

Seven women miscarried spontaneously at 4, 9, 7, 15, 13, 16 and 3 days after the C×1 measurement. The shortest endocervical length in these women was 3, 9, 21, 22, 28, 29 and 41 mm. No woman miscarried after C×2.

## Discussion

### Main findings

In our study population of mainly white women with a low prevalence of sPTB, the shorter the cervix in the second trimester the higher the likelihood of sPTB. All measurements, except isthmus length, had a similar ability to discriminate between women who did and did not give birth spontaneously at <33 weeks of gestation. The ability to discriminate was substantially better when measurements were taken at 21–23 weeks of gestation than at 18–20 weeks of gestation and was better the earlier in gestation the sPTB occurred. A change in cervical length between two measurements did not have discriminative ability superior to that of a single measurement.

### Strengths and limitations

Our study is the largest and most comprehensive blinded study describing the diagnostic performance of mid‐trimester sonographic cervical length to predict PTB. Blinding is essential for an estimation of the true association between the test results and the outcome^24^. Other strengths are few interventions (progesterone treatment or cerclage) after inclusion (Table S2), minimal loss to follow‐up, detailed description of measurement technique, rigorous quality control of the cervical length measurements, comparison of the discriminative ability between different measurements and comparison of performance between centres. Moreover, our clear description of patient flow and comparison of background and outcome data between study participants, decliners and a Swedish background population makes it possible to estimate selection bias. It is a limitation that our study populations include a slightly higher proportion of women at increased risk of sPTB than our control populations, and that sPTB was slightly more common in our study populations than in our control populations. On the other hand, it is likely that women who perceive themselves at increased risk are those that are the most likely to accept cervical length screening, should such screening be implemented. Another limitation is that not all women were assessed for eligibility, that 46% of eligible women declined cervix measurement and that only 54% (6179/11 456) of the participants underwent both a C×1 and a C×2 measurement. We chose PTB at <33 weeks of gestation as the primary outcome, as opposed to sPTB at <33 weeks of gestation, because classifying a delivery as spontaneous or indicated may be difficult. Our secondary outcomes are more clinically relevant because sPTB is potentially detectable with cervical length screening and is potentially preventable.^7^

### Interpretation

We identified five blinded studies reporting the sensitivity and specificity of second‐trimester sonographic cervical length for predicting sPTB in a general population of asymptomatic women with a singleton pregnancy.^6^, ^11^, ^12^, ^13^, ^14^ All but one are single‐centre studies.^6^ None provides a flow chart describing patient selection or compares the demographic characteristics of the study population with those of the general population of singleton pregnancies, even though Iams et al. state that ‘the study population was selected to reflect the parity and race of women receiving prenatal care at the participating centres’.^6^ The study populations vary in size from 529 to 3694, and the number of sPTBs used to calculate sensitivity is 10, 16, 19, 31 and 123. The studies differ substantially with regard to race and ethnicity (99% white,^14^ 63% black,^6^ 65% non‐white,^11^ 100% Chinese^13^ or not described^12^), outcome measures (sPTB at <35, <34 or <33 weeks of gestation) and prevalence of sPTB (<35 weeks of gestation, 0.8%,^14^ 1.3%^12^ or 4.3%^6^; <34 weeks of gestation, 0.7%^13^; <37 weeks of gestation, 4.3%^11^). These differences are likely to explain a substantial proportion of the disparate results: the prevalence of a shortest endocervical length of ≤25 mm ranges from 0.3%^14^ to 10.0%^6^; the sensitivity for predicting sPTB at <35 weeks of gestation when using the 25‐mm cut‐off varies from 7%^14^ to 37%^6^; and the specificity varies from 92 to 100%.

The aim of second‐trimester cervical length screening is to reduce the number of PTBs by offering prophylaxis to women identified as being at high risk. As only a proportion of PTBs are spontaneous (Table S2), and because a short cervix cannot predict indicated PTB, one cannot expect screening followed by prophylaxis to reduce the total number of PTBs dramatically. If we assume that 50% of singleton births at <33 weeks of gestation are spontaneous (Table S2), a screening method with 100% acceptance rate and 50% sensitivity to detect sPTB at <33 weeks of gestation could potentially result in 7.5% reduction in the total number of births at <33 weeks of gestation (provided that most women identified as high risk accept progesterone prophylaxis and that prophylaxis does indeed reduce the number of sPTBs by 30%).^7^ The percentage reduction in the number of PTBs at 33–36 weeks of gestation would be smaller, because of the poor ability of short cervix in the second trimester to predict sPTB at ≥ 33 weeks of gestation. Even if as many as 60 or 70% of PTBs were spontaneous,^25^ the effect of screening on the total number of PTBs would be small. The higher the detection rate the greater the potential of screening to reduce the number of PTBs, however.

The potential benefit of screening must be balanced against the workload imposed by screening and the potential negative effects of false‐positive screening results (unnecessary anxiety, unnecessary follow‐up examinations, unnecessary sick leave and unnecessary progesterone treatment). What is an acceptable balance between the positive and negative effects of screening and what is an acceptable workload is subjective and depends on the conditions under which one works. In our study, screening at 18–20 weeks of gestation did not result in a reasonable balance (Table 1). Screening at 18–20 weeks of gestation followed by progesterone prophylaxis could potentially prevent some late miscarriages, but it could also result in an increased number of extremely preterm births should progesterone treatment delay delivery by only a few weeks. Some might question whether the 25‐mm cut‐off for endocervical length at 21–23 weeks of gestation to predict sPTB at <33 weeks of gestation was associated with an acceptable balance between detection rate (38.5%), size of high‐risk group (4.4%), potential reduction in total number of PTBs at <33 weeks of gestation (5.5%), NNS to detect one sPTB at <33 weeks of gestation (*n* = 629), NNS to potentially prevent one sPTB at <33 weeks of gestation (*n* = 629/0.3 = 2097) and FP/TP (26). A cut‐off with higher detection rate might be preferable. The 27‐mm cut‐off (best cut‐off) had a higher detection rate (53.8%) and lower NNS to detect one sPTB at <33 weeks of gestation (449); however, it was associated with a larger high‐risk group (8.1%) and a higher FP/TP (35). Moreover, it has not been prospectively validated in another population similar to ours, and the effect of vaginal progesterone in women with a cervix of 26 or 27 mm in length is insufficiently known.^10^

### Conclusion

Second‐trimester sonographic cervical length can identify women at high risk of sPTB, but in a population of mainly white women with a low prevalence of sPTB its diagnostic performance is moderate. A health economic analysis using our results is justified.

### Disclosure of interests

None declared. Completed disclosure of interest forms are available to view online as supporting information.

### Contribution to authorship

LV, BJ, HH and UBW conceived and designed the study. PK, BJ, PL, HF, JW, UBW and LV oversaw recruitment and the examination of study participants and the collection of data at the local centres. LV, UBW, BJ, HH and PK wrote the statistical analysis plan together with two statisticians (Mattias Molin and Nils‐Gunnar Person, Statistical Consulting Group, Gothenburg). PK, UBW and LV performed the data cleaning together with statistician Mattias Molin. PK, LV, UBW, BJ, HH, PL, HF and JW interpreted the data. PK, LV and UBW wrote the first draft of the article, which was then critically reviewed and revised by the other co‐authors. All authors approved the final version of the article for submission. All authors had full access to all of the data in the study. LV and UBW are the guarantors. They take responsibility for the integrity of the data and the accuracy of the data analysis. The corresponding author (LV) had final responsibility for the decision to submit for publication and attests that all authors meet the criteria for authorship and that no others meeting the criteria have been omitted. UBW and LV contributed equally (last authors).

### Details of ethics approval

The study was approved by the Regional Ethical Review Board in Gothenburg (Dnr: 825‐13, 13 November 2013; T053‐14, 17 January 2014; T691‐14, 25 September 2014; T972‐15, 7 December 2015; T122‐16, 25 February 2016; T896‐17, 17 October 2017; T645‐18, 13 July 2018, T878‐18, 11 October 2018; T970‐18, 6 November 2018). All participants gave written informed consent before taking part in the study.

### Funding

The study was funded by The Swedish Research Council (Dnr 2014‐06998), Forskning och Utbildning (FoU) Södra Älvsborg, by the Swedish state under the agreement between the Swedish Government and the County Councils, the ALF‐agreement (ALFGBG‐136431, ALFGBG‐426411, ALFGBG‐71859) and The Swedish National Patient Insurance Company (LÖF). The funders had no role in the study design, in the collection, analysis and interpretation of data, in the writing of the report and in the decision to submit the work for publication. The researchers were independent of the funders.

### Acknowledgements

Mattias Molin, Statistical Consulting Group, Gothenburg, performed the statistical analyses. Eva Hessman, librarian at the Biomedical Library, helped with the literature search. Agneta Cedefors‐Blom helped with administration.

## Supporting information


**Figure S1.** Kaplan–Meier plot showing the proportion of women still pregnant at different gestational ages before 37^+0^ weeks of gestation in relation to shortest endocervical length at 18^+0^–20^+6^ weeks of gestation (C×1).

**Figure S2.** Kaplan–Meier plot showing the proportion of women still pregnant at different gestational ages before 37^+0^ weeks of gestation in relation to shortest endocervical length at 21^+0^–23^+6^ weeks of gestation (C×2).

**Table S1.** Demographics and baseline characteristics by study groups.

**Table S2.** Pregnancy, delivery and neonatal outcome by study groups.

**Table S3.** Area under the receiver operating characteristic curve for different cervical length measurements with regards to spontaneous preterm birth (including late miscarriage at 18^+0^–21^+6^ weeks of gestation) for the three study populations with cervical measurements at 18^+0^–20^+6^ weeks of gestation (C×1), at 21^+0^–23^+6^ weeks of gestation (C×2) and both at C×1 and C×2 (C×1 C×2).

**Table S4.** Area under the receiver operating characteristic curve for different cervical length measurements with regards to preterm birth at <33 weeks of gestation excluding late miscarriage at 18^+0^–20^+6^ weeks of gestation (primary outcome), and with regards to preterm birth including late miscarriage at 18^+0^–20^+6^ weeks of gestation, for the three study populations with cervical measurements at 18^+0^–20^+6^ weeks of gestation (C×1), at 21^+0^–23^+6^ weeks of gestation (C×2), and both at C×1 and C×2 (C×1 C×2).

**Table S5.** Area under the receiver operating characteristic curve for different cervical length measurements at 18^+0^–20^+6^ weeks of gestation (C×1, *n* = 11 072) for prediction of preterm birth or spontaneous preterm birth at <32, <33 and <34 weeks of gestation in women with, versus without, isthmus.

**Table S6.** Shortest endocervical length (distance A–B) at 18^+0^–20^+6^ weeks of gestation (C×1) and at 21^+0^–23^+6^ weeks of gestation (C×2).

**Table S7.** Area under the receiver operating characteristic curve for different cervical length measurements with regards to preterm birth at <33 weeks of gestation excluding late miscarriage at 18^+0^–20^+6^ weeks of gestation (primary outcome), and with regards to spontaneous preterm birth including late miscarriage at 18^+0^–20^+6^ weeks of gestation, for the study population with two cervical measurements at 18^+0^–20^+6^ weeks of gestation and at 21^+0^–23^+6^ weeks of gestation, with at least 14 days between the two measurements (C×1 C×2).

**Table S8.** Discriminative ability in terms of positive and negative likelihood ratios, and positive and negative predictive values, for shortest endocervical length (distance A–B) measured at 18^+0^–20^+6^ weeks of gestation (C×1, *n* = 11 072) with regards to predicting spontaneous preterm birth, including late spontaneous miscarriage.

**Table S9.** Discriminative ability in terms of positive and negative likelihood ratios, and positive and negative predictive values, for shortest endocervical length (distance A–B) measured at 21^+0^–23^+6^ weeks of gestation (C×2, *n* = 6288) with regards to predicting spontaneous preterm birth.

**Table S10.** Discriminative ability of shortest endocervical length (distance A–B) of 0–30 mm at 18^+0^–20^+6^ weeks of gestation (C×1, *n* = 11 072) with regards to predicting spontaneous preterm birth, including late spontaneous miscarriage.

**Table S11.** Discriminative ability of shortest endocervical length (distance A–B) of 0–30 mm at 21^+0^–23^+6^ weeks of gestation (C×2, *n* = 6288) with regards to predicting spontaneous preterm birth.

Supplementary Material

Supplementary Material

Supplementary Material

Supplementary Material

Supplementary Material

Supplementary Material

Supplementary Material

Supplementary Material

## Data Availability

Data available from the corresponding author upon reasonable request.
