# Supplementary material for: Second‐trimester transvaginal ultrasound measurement of cervical length for prediction of preterm birth: a blinded prospective multicentre diagnostic accuracy study
Source: BJOG. 2020 Oct 19;128(2):195–206. doi: 10.1111/1471-0528.16519 (PMC7821210; doi:10.1111/1471-0528.16519)
Supplement: Supplementary file 1 — Figure S1. Kaplan–Meier plot showing the proportion of women still pregnant at different gestational ages before 37+0 weeks of gestation in relation to shortest endocervical length at 18+0–20+6 weeks of gestation (C×1). [file BJO-128-195-s001.pdf]

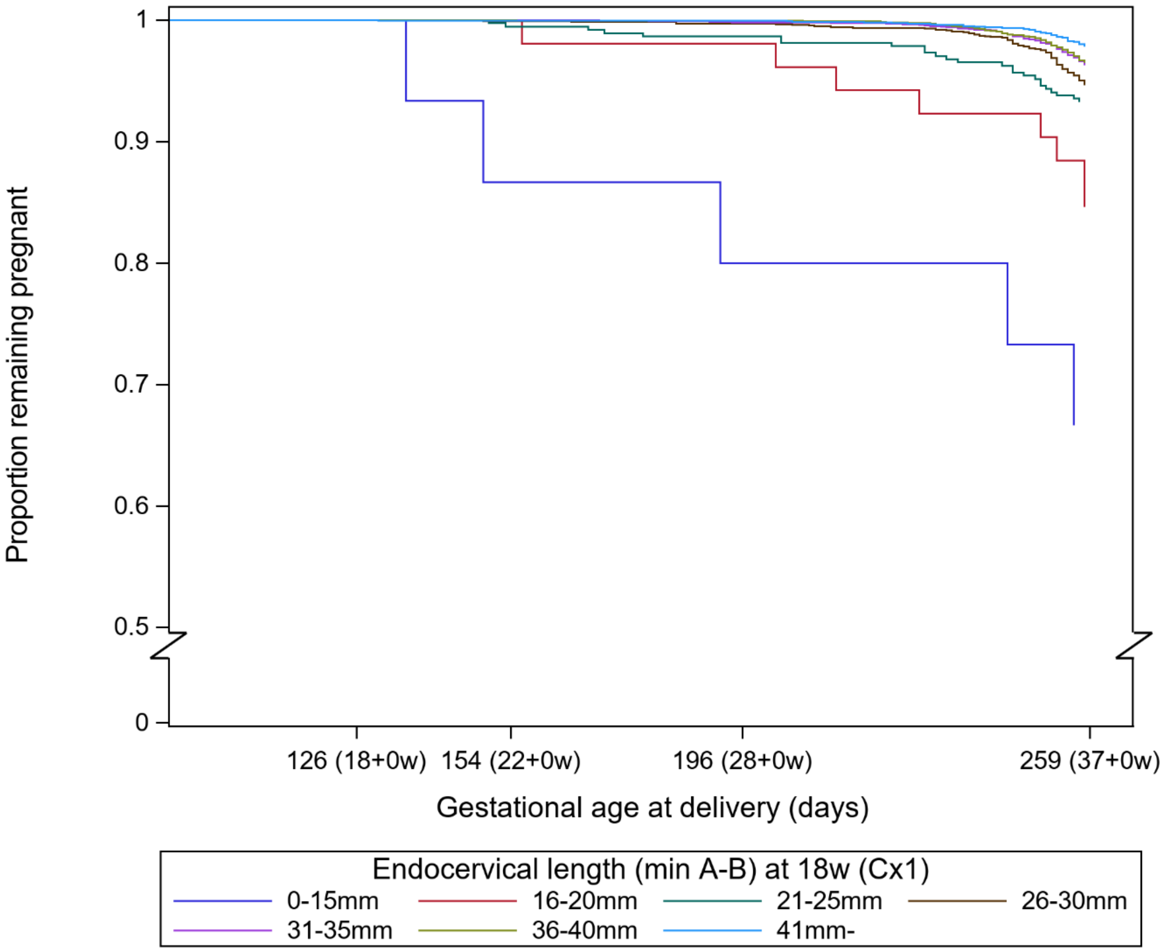

Number at risk

41mm-	2298	2297	2296	2210
36-40mm	3355	3355	3350	3197
31-35mm	3244	3243	3232	3072
26-30mm	1734	1732	1726	1611
21-25mm	374	372	368	343
16-20mm	52	52	51	44
0-15mm	15	13	12	10

Figure S1. Kaplan-Meier plot showing the proportion of women still pregnant at different gestational ages before 37+0 weeks in relation to shortest endocervical length at 18 weeks+0 days to 20 weeks+6 days (Cx1). Women with indicated preterm delivery are censored. w = gestational weeks.
