# Supplementary material for: Second‐trimester transvaginal ultrasound measurement of cervical length for prediction of preterm birth: a blinded prospective multicentre diagnostic accuracy study
Source: BJOG. 2020 Oct 19;128(2):195–206. doi: 10.1111/1471-0528.16519 (PMC7821210; doi:10.1111/1471-0528.16519)
Supplement: Supplementary file 2 — Figure S2. Kaplan–Meier plot showing the proportion of women still pregnant at different gestational ages before 37+0 weeks of gestation in relation to shortest endocervical length at 21+0–23+6 weeks of gestation (C×2). [file BJO-128-195-s002.pdf]

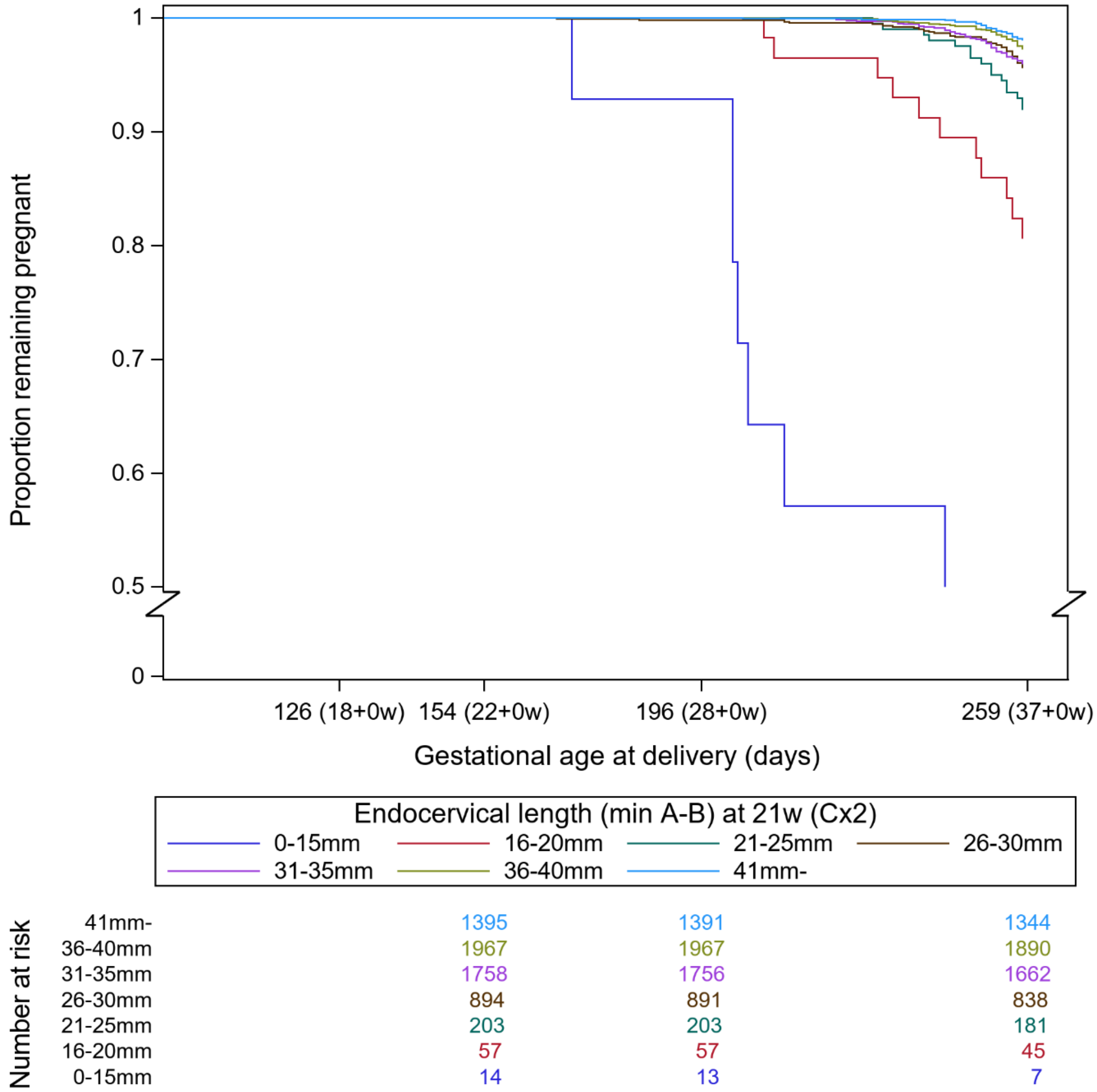

Figure S2. Kaplan-Meier plot showing the proportion of women still pregnant at different gestational ages before 37+0 weeks in relation to shortest endocervical length at 21 weeks+0 days to 23 weeks+6 days (Cx2). Women with indicated preterm delivery are censored. w =gestational weeks.
