# Supplementary material for: Second‐trimester transvaginal ultrasound measurement of cervical length for prediction of preterm birth: a blinded prospective multicentre diagnostic accuracy study
Source: BJOG. 2020 Oct 19;128(2):195–206. doi: 10.1111/1471-0528.16519 (PMC7821210; doi:10.1111/1471-0528.16519)
Supplement: Supplementary file 3 — Table S1. Demographics and baseline characteristics by study groups. [file BJO-128-195-s003.pdf]

Table S1. Demographics and baseline characteristics by study groups

	Study groups				
	Cx1 (measurement at 18+0-20+6 GW) (n=11072)	Cx2 (measurement at 21+0-23+6 GW) (n=6288)	Cx1Cx2 (measurement at 18+0-20+6 and 21+0-23+6 GW) (n=6179)	No cervix measurement (decliners) (n=9799)	Swedish background population (n=347 479)
Age at delivery (years)	31.5 (4.7)<br>31.3 (28.3; 34.8)<br>n=11 072	31.5 (4.6)<br>31.3 (28.3; 34.6)<br>n=6288	31.4 (4.6)<br>31.3 (28.3; 34.6)<br>n=6179	31.0 (4.7)<br>30.9 (27.7; 34.3)<br>n=9797	30.8 (5.2)<br>30.7 (27.1; 34.4)<br>n=347 479
Ethnicity (self-reported)					
White	9898 (89.4%)	5796 (92.2%)	5696 (92.2%)	8793 (89.8%)	NA
Black	255 (2.3%)	105 (1.7%)	102 (1.7%)	192 (2.0%)	NA
Mixed White-Black	48 (0.4%)	19 (0.3%)	19 (0.3%)	45 (0.5%)	NA
Middle East	405 (3.7%)	157 (2.5%)	155 (2.5%)	477 (4.9%)	NA
Indian	116 (1.0%)	51 (0.8%)	50 (0.8%)	72 (0.7%)	NA
Southeast Asian	218 (2.0%)	101 (1.6%)	100 (1.6%)	126 (1.3%)	NA
Other	132 (1.2%)	59 (0.9%)	57 (0.9%)	87 (0.9%)	NA
Maternal country of birth					
Sweden	8309 (81.7%)	4940 (84.6%)	4855 (84.6%)	7550 (83.9%)	227 270 (73.2%)
Other Nordic countries	141 (1.4%)	80 (1.4%)	79 (1.4%)	80 (0.9%)	2907 (0.9%)
Other European countries	717 (7.0%)	391 (6.7%)	382 (6.7%)	520 (5.8%)	23 744 (7.6%)
Outside European countries	1008 (9.9%)	426 (7.3%)	420 (7.3%)	854 (9.5%)	56 530 (18.2%)
Highest level of education					
<9 years	33 (0.3%)	10 (0.2%)	10 (0.2%)	48 (0.6%)	4674 (1.7%)
9 years	304 (3.2%)	148 (2.7%)	145 (2.7%)	286 (3.4%)	19 496 (6.9%)
12 years	3238 (33.8%)	1880 (33.9%)	1853 (34.0%)	3313 (39.2%)	111 301 (39.4%)
>12 years	6016 (62.7%)	3512 (63.3%)	3443 (63.2%)	4808 (56.9%)	147 193 (52.1%)
Main occupation					
Employed	8242 (81.0%)	4860 (83.2%)	4770 (83.1%)	7224 (80.3%)	225 225 (72.6%)
Student	799 (7.9%)	399 (6.8%)	395 (6.9%)	612 (6.8%)	35 114 (11.3%)
Maternity leave	499 (4.9%)	239 (4.1%)	236 (4.1%)	571 (6.4%)	13 915 (4.5%)
Unemployed	282 (2.8%)	144 (2.5%)	142 (2.5%)	285 (3.2%)	13 035 (4.2%)
Sick leave	232 (2.3%)	145 (2.5%)	144 (2.5%)	197 (2.2%)	6522 (2.1%)
Other	117 (1.2%)	53 (0.9%)	52 (0.9%)	102 (1.1%)	16293 (5.3%)
Height at first antenatal visit (cm)	166.8 (6.4)<br>167.0<br>(163; 171)<br>n=10 157	167.0 (6.3)<br>167.0<br>(163; 171)<br>n=5829	167.0 (6.3)<br>167.0<br>(163; 171)<br>n=5730	166.9 (6.3)<br>167.0<br>(163; 171)<br>n=8677	165.9 (6.6)<br>166.0<br>(162; 170)<br>n=327 657
BMI at first antenatal visit	24.7 (4.5)<br>23.7<br>(21.6; 26.8)<br>n=9968	24.8 (4.5)<br>23.8<br>(21.6; 26.9)<br>n=5730	24.8 (4.5)<br>23.8<br>(21.6; 26.9)<br>n=5634	24.8 (4.7)<br>23.7<br>(21.5; 27.0)<br>n=8530	24.8 (4.7)<br>23.8<br>(21.5; 27.1)<br>n=322 186
Smoking and/or using snuff at first antenatal visit	484 (4.4%)	243 (3.9%)	239 (3.9%)	465 (4.8%)	19 540 (5.6%)
Smoking at first antenatal visit	390 (3.9%)	189 (3.3%)	186 (3.3%)	375 (4.5%)	15 869 (4.7%)
Alcohol screening performed*(AUDIT)	8622 (77.9%)	4824 (76.7%)	4730 (76.5%)	7308 (74.6%)	273 862 (78.8%)

AUDIT score ≥6 (dangerous use of alcohol)	518 (6.0%)	298 (6.2%)	294 (6.2%)	378 (5.2%)	13 547 (4.9%)
IVF in current pregnancy	587 (5.5%)	359 (5.9%)	350 (5.9%)	376 (4.0%)	15 248 (4.6%)
Chronic hypertension registered at first antenatal visit	52 (0.5%)	27 (0.4%)	26 (0.4%)	46 (0.5%)	1574 (0.5%)
Diabetes mellitus type 1 or 2 registered at first antenatal visit	97 (0.9%)	51 (0.8%)	50 (0.8%)	71 (0.8%)	2673 (0.8%)
Renal disease registered at first antenatal visit	56 (0.5%)	33 (0.5%)	33 (0.6%)	43 (0.5%)	1525 (0.5%)
Conization of cervix before pregnancy	657 (5.9%)	404 (6.4%)	397 (6.4%)	418 (4.2%)	14 720 (4.3%) n=340 999
Number of previous pregnancies					
0	3963 (35.8%)	2316 (36.8%)	2275 (36.8%)	3401 (34.7%)	NA
1	3367 (30.4%)	1962 (31.2%)	1929 (31.2%)	3028 (30.9%)	NA
≥2	3742 (33.8%)	2010 (32.0%)	1975 (32.0%)	3370 (34.4%)	NA
Previous stillbirth ≥1	42 (0.4%)	21 (0.3%)	19 (0.3%)	40 (0.4%)	2053 (0.6%)
Parity					
0	5223 (49.5%)	3131 (51.6%)	3084 (51.7%)	4175 (45.2%)	162 244 (48.0%)
1	3756 (35.6%)	2083 (34.3%)	2038 (34.2%)	3516 (38.0%)	110 419 (32.6%)
≥2	1577 (14.9%)	857 (14.1%)	845 (14.1%)	1551 (16.8%)	65 640 (19.4%)
Previous PTB (<37 GW) ≥1†	498 (4.5%)	301 (4.8%)	293 (4.8%)	301 (3.1%)	11 360 (3.3%) n=340 999
Previous singleton spontaneous PTB <37 GW ≥1	360 (3.3%)	216 (3.5%)	214 (3.5%)	196 (2.1%)	7860 (2.3%) n=340 999
Previous singleton spontaneous PTB <33 GW ≥1	64 (0.6%)	33 (0.5 %)	32 (0.5%)	22 (0.3%)	1290 (0.4%) n=340 999

For categorical variables n (%) is presented
For continuous variables Mean (SD) / Median / (25th percentile; 75th centile) /n is presented

AUDIT=Alcohol Use Disorder Test. BMI=body mass index (kg/m^2), GW=gestational weeks, IVF=in vitro fertilization, PTB=preterm birth, NA=not available

* alcohol screening by AUDIT tool according to antenatal care routines[1]

† previous singleton, multiple, spontaneous and indicated **PTBs** are included

Reference

[1]Göransson M, Magnusson A, Heilig M. Identifying hazardous alcohol consumption during pregnancy: implementing a research-based model in real life. *Acta Obstet Gynecol Scand.* 2006;85:657-62
