# Supplementary material for: Second‐trimester transvaginal ultrasound measurement of cervical length for prediction of preterm birth: a blinded prospective multicentre diagnostic accuracy study
Source: BJOG. 2020 Oct 19;128(2):195–206. doi: 10.1111/1471-0528.16519 (PMC7821210; doi:10.1111/1471-0528.16519)
Supplement: Supplementary file 4 — Table S2. Pregnancy, delivery and neonatal outcome by study groups. [file BJO-128-195-s004.pdf]

Table S2. Pregnancy, delivery and neonatal outcome by study groups

Variable	Study groups				
	Cx1 (measurement at 18+0-20+6 GW) (n=11072)	Cx2 (measurement at 21+0-23+6 GW) (n=6288)	Cx1Cx2 (measurement at 18+0-20+6 and 21+0-23+6 GW) (n=6179)	No cervix measurement (decliners) (n=9799)	Swedish background population (n=347 479)
Redeemed prescription of vaginal progesterone after inclusion/18+0 GW until delivery	18 (0.2%)	9 (0.1%)	8 (0.1%)	0 (0.0%)	823 (0.2%)<br>n=340 999
Cerclage after inclusion/18+0 GW*	1 (0.0%)	0 (0.0%)	0 (0.0%)	0 (0.0%)	72 (0.0%)<br>n=340 999
Preeclampsia or gestational hypertension at delivery	619 (5.6%)	368 (5.9%)	359 (5.8%)	534 (5.4%)	16 988 (4.9%)
Chronic hypertension at delivery	73 (0.7%)	39 (0.6%)	37 (0.6%)	48 (0.5%)	1964 (0.6%)
Diabetes type 1, 2 or gestational diabetes at delivery	271 (2.4%)	171 (2.7%)	169 (2.7%)	201 (2.1%)	7054 (2.0%)
Induction of labour	1946 (17.6%)	1090 (17.3%)	1066 (17.3%)	1591 (16.2%)	59 360 (17.1%)
Caesarean delivery	1704 (15.5%)	944 (15.1%)	923 (15.0%)	1475 (15.2%)	60 113 (17.3%)
Gestational age at birth (days)	277.8 (13.2)<br>280.0<br>(273; 286)<br>n=11 072	278.0 (12.2)<br>280.0<br>(273; 286)<br>n=6288	278.0 (12.1)<br>280.0<br>(273; 286)<br>n=6179	278.4 (12.4)<br>280.0<br>(273; 286)<br>n=9799	277.9 (13.2)<br>280.0<br>(273; 286)<br>n=347 479
Late miscarriage† (before 22+0 GW)	8 (0.1%)	0 (0.0%)	0 (0.0%)	7 (0.1%)	NA
Spontaneous late miscarriage (before 22+0 GW)	7 (0.1%)	0 (0.0%)	0 (0.0%)	4 (0.0%)	NA
PTB <33 GW‡	109 (1.0%)	53 (0.8%)	52 (0.8%)	72 (0.7%)	3767 (1.1%)
PTB <37 GW‡	577 (5.2%)	321 (5.1%)	313 (5.1%)	412 (4.2%)	18 108 (5.2%)
Spontaneous PTB <28 GW‡	15 (0.1%)	3 (0.0%)	3 (0.0%)	18 (0.2%)	682 (0.2%)
Spontaneous PTB <29 GW‡	17 (0.2%)	5 (0.1%)	5 (0.1%)	19 (0.2%)	816 (0.2%)
Spontaneous PTB <30 GW‡	27 (0.2%)	10 (0.2%)	10 (0.2%)	25 (0.3%)	993 (0.3%)
Spontaneous PTB <31 GW‡	33 (0.3%)	15 (0.2%)	15 (0.2%)	30 (0.3%)	1224 (0.4%)
Spontaneous PTB <32 GW‡	39 (0.4%)	18 (0.3%)	18 (0.3%)	36 (0.4%)	1521 (0.4%)
Spontaneous PTB <33 GW‡	56 (0.5%)	26 (0.4%)	26 (0.4%)	42 (0.4%)	1958 (0.6%)
Spontaneous PTB <34 GW‡	87 (0.8%)	41 (0.7%)	40 (0.6%)	58 (0.6%)	2732 (0.8%)
Spontaneous PTB <35 GW‡	136 (1.2%)	69 (1.1%)	67 (1.1%)	102 (1.0%)	4038 (1.2%)
Spontaneous PTB <36 GW‡	219 (2.0%)	114 (1.8%)	109 (1.8%)	164 (1.7%)	6324 (1.8%)
Spontaneous PTB <37 GW‡	410 (3.7%)	225 (3.6%)	220 (3.6%)	293 (3.0%)	10 967 (3.2%)
Birth weight (g)	3556 (548)<br>3570<br>(3240; 3905)<br>n=10 915	3569 (539)<br>3580<br>(3250; 3915)<br>n=6207	3569 (539)<br>3582<br>(3250; 3915)<br>n=6099	3584 (538)<br>3590<br>(3270; 3925)<br>n=9704	3518 (566)<br>3530<br>(3200; 3870)<br>n=346 241
Small for gestational age§	285 (2.6%)	139 (2.2%)	138 (2.2%)	243 (2.5%)	8319 (2.4%)
Apgar score at 5 min <7	155 (1.4%)	91 (1.4%)	89 (1.4%)	125 (1.3%)	5228 (1.6%)
Malformations¶	511 (4.6%)	282 (4.5%)	274 (4.4%)	414 (4.2%)	14 839 (4.3%)
Perinatal mortality**	38 (0.3%)	17 (0.3%)	17 (0.3%)	26 (0.3%)	1385 (0.4%)
Stillbirth after 22+0 GW	32 (0.3%)	15 (0.2%)	15 (0.2%)	24 (0.2%)	1183 (0.3%)
Neonatal death within 7 days after birth	6 (0.1%)	2 (0.0%)	2 (0.0%)	2 (0.0%)	203 (0.1%)

For categorical variables n (%) is presented
For continuous variables Mean (SD) / Median / (25th percentile; 75th percentile) / n is presented
GW=gestational weeks, PTB=preterm birth
* cerclage from 18+0 GW for Swedish background population
† includes one case of induction of labour for missed abortion
‡ PTB and spontaneous PTB include births from 22+0 GW
§ defined as ≥2 SDs below the Swedish gestational age- and sex-specific growth standard[1]
¶ malformation defined as a newborn with an International Classification of Diseases (ICD) -10 Q diagnosis registered at birth
** includes stillbirth after 22+0 GW and neonatal death within 7 days after birth

Reference

1. Marsál K, Persson PH, Larsen T, Lilja H, Selbing A, Sultan B. Intrauterine growth curves based on ultrasonically estimated foetal weights. *Acta Paediatr*. 1996;85:843-8
