# Supplementary material for: Second‐trimester transvaginal ultrasound measurement of cervical length for prediction of preterm birth: a blinded prospective multicentre diagnostic accuracy study
Source: BJOG. 2020 Oct 19;128(2):195–206. doi: 10.1111/1471-0528.16519 (PMC7821210; doi:10.1111/1471-0528.16519)
Supplement: Supplementary file 5 — Table S3. Area under the receiver operating characteristic curve for different cervical length measurements with regards to spontaneous preterm birth (including late miscarriage at 18+0–21+6 weeks of gestation) for the three study populations with cervical measurements at 18+0–20+6 weeks of gestation (C×1), at 21+0–23+6 weeks of gestation (C×2) and both at C×1 and C×2 (C×1 C×2). [file BJO-128-195-s005.pdf]

Table S3. Area under the Receiver Operating Characteristic curve for different cervical length measurements with regard to spontaneous preterm birth including late miscarriage at 18+0 to 21+6 weeks for the three study populations with cervical measurements at 18+0 to 20+6 weeks (Cx1), at 21+0 to 23+6 weeks (Cx2), and both at Cx1 and Cx2 (Cx1Cx2)

	**Area under the Receiver Operating Characteristic curve (95% confidence interval)**									
**Cervical measurement***	**sPTB <28 GW**	**sPTB <29 GW**	**sPTB <30 GW**	**sPTB <31 GW**	**sPTB <32 GW**	**sPTB <33 GW**	**sPTB <34 GW**	**sPTB <35 GW**	**sPTB <36 GW**	**sPTB <37 GW**
**Cx1 (n=11 072)**	**n=22**	**n=24**	**n=34**	**n=40**	**n=46**	**n=63**	**n=94**	**n=143**	**n=226**	**n=417**
Min A-B (Cx1)	0.83	0.84	0.77	0.76	0.71	0.68	0.65	0.62	0.61	0.60
	(0.74; 0.92)	(0.76; 0.92)	(0.68; 0.87)	(0.67; 0.85)	(0.62; 0.80)	(0.60; 0.76)	(0.59; 0.71)	(0.57; 0.67)	(0.57; 0.65)	(0.57; 0.63)
Mean A-B (Cx1)	0.82	0.83	0.77	0.75	0.71	0.68	0.65	0.62	0.61	0.60
	(0.73; 0.90)	(0.75: 0.91)	(0.68; 0.86)	(0.67; 0.84)	(0.62; 0.80)	(0.61; 0.76)	(0.59; 0.71)	(0.57; 0.66)	(0.57; 0.65)	(0.57; 0.62)
Max A-B (Cx1)	0.80	0.81	0.75	0.74	0.69	0.67	0.65	0.61	0.60	0.59
	(0.71; 0.89)	(0.73; 0.89)	(0.66; 0.84)	(0.65; 0.82)	(0.61; 0.78)	(0.60; 0.75)	(0.59; 0.71)	(0.57; 0.66)	(0.57; 0.64)	(0.56; 0.62)
Min A-C (Cx1)	0.83	0.84	0.77	0.76	0.71	0.69	0.65	0.62	0.60	0.59
	(0.73; 0.92)	(0.75; 0.93)	(0.68; 0.86)	(0.68; 0.85)	(0.62; 0.81)	(0.61; 0.77)	(0.59; 0.72)	(0.57; 0.67)	(0.56; 0.64)	(0.56; 0.62)
Mean A-C (Cx1)	0.82	0.84	0.77	0.76	0.71	0.69	0.66	0.62	0.60	0.59
	(0.73; 0.92)	(0.75; 0.93)	(0.68; 0.86)	(0.68; 0.85)	(0.62; 0.80)	(0.61; 0.77)	(0.59; 0.72)	(0.57; 0.67)	(0.56; 0.64)	(0.56; 0.62)
Max A-C (Cx1)	0.82	0.83	0.76	0.76	0.71	0.69	0.66	0.62	0.60	0.59
	(0.72; 0.91)	(0.74; 0.92)	(0.67; 0.85)	(0.67; 0.84)	(0.61; 0.80)	(0.61; 0.77)	(0.59; 0.72)	(0.57; 0.67)	(0.56; 0.64)	(0.59; 0.62)
Min A-B+B-C (Cx1)	0.83	0.84	0.77	0.77	0.71	0.69	0.65	0.61	0.59	0.59
	(0.73; 0.93)	(0.75; 0.93)	(0.68; 0.87)	(0.68; 0.95)	(0.62; 0.81)	(0.61; 0.77)	(0.59; 0.72)	(0.56; 0.66)	(0.55; 0.63)	(0.56; 0.62)
Mean A-B+B-C (Cx1)	0.82	0.83	0.76	0.76	0.71	0.69	0.65	0.61	0.59	0.59
	(0.72; 0.91)	(0.74; 0.92)	(0.67; 0.86)	(0.67; 0.84)	(0.61; 0.80)	(0.61; 0.77)	(0.59; 0.72)	(0.56; 0.66)	(0.55; 0.63)	(0.56; 0.61)
Max A-B+B-C (Cx1)	0.78	0.80	0.74	0.74	0.69	0.68	0.64	0.60	0.58	0.58
	(0.67; 0.89)	(0.69; 0.90)	(0.64; 0.84)	(0.64; 0.83)	(0.59; 0.78)	(0.60; 0.76)	(0.58; 0.71)	(0.55; 0.65)	(0.54; 0.62)	(0.55; 0.61)
**Cx2 (n=6288)**	**n=3**	**n=5**	**n=10**	**n=15**	**n=18**	**n=26**	**n=41**	**n=69**	**n=114**	**n=225**
Min A-B (Cx2)	0.96	0.98	0.86	0.85	0.81	0.76	0.71	0.71	0.66	0.63
	(0.92; 1,00)	(0.95; 1.00)	(0.69; 1.00)	(0.71; 0.99)	(0.68; 0.93)	(0.65; 0.87)	(0.63; 0.80)	(0.65; 0.77)	(0.61; 0.72)	(0.59; 0.67)
Mean A-B (Cx2)	0.96	0.98	0.87	0.85	0.81	0.77	0.72	0.71	0.66	0.63
	(0.93; 1.00)	(0.95; 1.00)	(0.69; 1.00)	(0.72; 0.99)	(0.69; 0.93)	(0.66; 0.87)	(0.64; 0.80)	(0.65; 0.77)	(0.61; 0.71)	(0.59; 0.67)
Max A-B (Cx2)	0.96	0.98	0.87	0.86	0.82	0.77	0.73	0.71	0.66	0.62
	(0.92; 1.00)	(0.95; 1.00)	(0.70; 1.00)	(0.73; 0.99)	(0.70; 0.93)	(0.67; 0.87)	(0.65; 0.81)	(0.64; 0.77)	(0.61; 0.71)	(0.58; 0.66)
Min A-C (Cx2)	0.96	0.98	0.87	0.86	0.82	0.78	0.73	0.71	0.66	0.63
	(0.93; 1.00)	(0.95; 1.00)	(0.71; 1.00)	(0.73; 0.99)	(0.71; 0.94)	(0.68; 0.88)	(0.66; 0.81)	(0.65; 0.78)	(0.61; 0.72)	(0.60; 0.67)
Mean A-C (Cx2)	0.96	0.98	0.88	0.86	0.83	0.78	0.74	0.71	0.66	0.63
	(0.93; 1.00)	(0.95; 1.00)	(0.71; 1.00)	(0.74; 0.99)	(0.71; 0.94)	(0.69; 0.88)	(0.67; 0.82)	(0.65; 0.78)	(0.61; 0.71)	(0.59; 0.67)

Max A-C (Cx2)	0.96<br>(0.93; 1.00)	0.98<br>(0.95; 1.00)	0.88<br>(0.72; 1.00)	0.87<br>(0.75; 0.99)	0.83<br>(0.72; 0.94)	0.79<br>(0.70; 0.88)	0.75<br>(0.67; 0.82)	0.71<br>(0.65; 0.78)	0.66<br>(0.60; 0.71)	0.63<br>(0.59; 0.67)
Min A-B+B-C (Cx2)	0.96<br>(0.93; 1.00)	0.98<br>(0.95; 1.00)	0.87<br>(0.71; 1.00)	0.86<br>(0.73; 0.99)	0.82<br>(0.71; 0.94)	0.78<br>(0.68; 0.88)	0.74<br>(0.66; 0.81)	0.71<br>(0.65; 0.78)	0.67<br>(0.61; 0.72)	0.63<br>(0.60; 0.67)
Mean A-B+B-C (Cx2)	0.97<br>(0.93; 1.00)	0.98<br>(0.96; 1.00)	0.88<br>(0.72; 1.00)	0.87<br>(0.74; 0.99)	0.83<br>(0.72; 0.94)	0.79<br>(0.69; 0.88)	0.74<br>(0.67; 0.82)	0.71<br>(0.65; 0.78)	0.66<br>(0.61; 0.71)	0.63<br>(0.59; 0.67)
Max A-B+B-C (Cx2)	0.96<br>(0.93; 1.00)	0.98<br>(0.95; 1.00)	0.88<br>(0.72; 1.00)	0.87<br>(0.75; 0.99)	0.83<br>(0.72; 0.94)	0.79<br>(0.70; 0.88)	0.75<br>(0.68; 0.82)	0.71<br>(0.65; 0.78)	0.66<br>(0.60; 0.71)	0.63<br>(0.59; 0.67)
**Cx1Cx2 (n=6179)**	**n=3**	**n=5**	**n=10**	**n=15**	**n=18**	**n=26**	**n=40**	**n=67**	**n=109**	**n=220**
Change Min A-B in mm between Cx1 and Cx2	0.54<br>(0.19; 0.90)	0.68<br>(0.41; 0.94)	0.71<br>(0.52; 0.91)	0.69<br>(0.52; 0.86)	0.69<br>(0.54; 0.84)	0.67<br>(0.55; 0.79)	0.60<br>(0.51; 0.70)	0.63<br>(0.56; 0.69)	0.58<br>(0.52; 0.63)	0.56<br>(0.52; 0.60)
Change Mean A-B in mm between Cx1 and Cx2	0.66<br>(0.25;1.00	0.75<br>(0.49; 1.00)	0.76<br>(0.56; 0.95)	0.72<br>(0.54; 0.89)	0.72<br>(0.57; 0.87)	0.68<br>(0.57; 0.80)	0.62<br>(0.51; 0.71)	0.63<br>(0.56; 0.70)	0.58<br>(0.52; 0.63)	0.55<br>(0.51; 0.59)
Change Max A-B in mm between Cx1 and Cx2	0.69<br>(0.26; 1.00)	0.77<br>(0.51; 1.00)	0.76<br>(0.58; 0.95)	0.72<br>(0.55; 0.89)	0.72<br>(0.57; 0.87)	0.68<br>(0.56; 0.80)	0.60<br>(0.51; 0.70)	0.62<br>(0.55; 0.69)	0.57<br>(0.51; 0.63)	0.55<br>(0.51; 0.59)
Change Min A-C in mm between Cx1 and Cx2	0.65<br>(0.35; 0.96)	0.71<br>(0.51; 0.91)	0.70<br>(0.53; 0.87)	0.67<br>(0.51; 0.82)	0.68<br>(0.54; 0.83)	0.65<br>(0.53; 0.77)	0.62<br>(0.52; 0.72)	0.61<br>(0.54; 0.68)	0.56<br>(0.50; 0.62)	0.54<br>(0.50; 0.58)
Change Mean A-C in mm between Cx1 and Cx2	0.73<br>(0.53; 0.93)	0.76<br>(0.63; 0.89)	0.73<br>(0.58; 0.88)	0.68<br>(0.53; 0.83)	0.70<br>(0.56; 0.83)	0.66<br>(0.54; 0.77)	0.62<br>(0.53; 0.71)	0.61<br>(0.54; 0.68)	0.56<br>(0.50; 0.62)	0.53<br>(0.49; 0.57)
Change Max A-C in mm between Cx1 and Cx2	0.76<br>(0.60; 0.92)	0.78<br>(0.67; 0.89)	0.73<br>(0.59; 0.88)	0.68<br>(0.54; 0.83)	0.69<br>(0.56; 0.83)	0.65<br>(0.54; 0.76)	0.61<br>(0.52; 0.71)	0.61<br>(0.54; 0.68)	0.55<br>(0.59; 0.61)	0.53<br>(0.49; 0.57)
Change Min A-B+B-C in mm between Cx1 and Cx2	0.65<br>(0.41; 0.89)	0.71<br>(0.54; 0.87)	0.70<br>(0.53; 0.86)	0.66<br>(0.51; 0.82)	0.68<br>(0.54; 0.82)	0.65<br>(0.53; 0.76)	0.62<br>(0.52; 0.71)	0.61<br>(0.54; 0.68)	0.56<br>(0.50; 0.62)	0.54<br>(0.50; 0.58)
Change Mean A-B+B-C in mm between Cx1 and Cx2	0.77<br>(0.60; 0.94)	0.78<br>(0.66; 0.90)	0.73<br>(0.58; 0.88)	0.68<br>(0.53; 0.83)	0.70<br>(0.56; 0.83)	0.66<br>(0.54; 0.77)	0.62<br>(0.52; 0.71)	0.61<br>(0.54; 0.68)	0.56<br>(0.50; 0.61)	0.53<br>(0.49; 0.57
Change Max A-B+B-C in mm between Cx1 and Cx2	0.81<br>(0.65; 0.97)	0.80<br>(0.69; 0.91)	0.74<br>(0.59; 0.88)	0.68<br>(0.54; 0.83)	0.69<br>(0.56; 0.83)	0.65<br>(0.54; 0.76)	0.61<br>(0.52; 0.71)	0.60<br>(0.53; 0.67)	0.55<br>(0.49; 0.61)	0.52<br>(0.48; 0.56)
% Change Min A-B between Cx1 and Cx2	0.55<br>(0.10; 1.00)	0.72<br>(0.40; 1.00)	0.74<br>(0.53; 0.96)	0.71<br>(0.53; 0.90)	0.71<br>(0.55; 0.87)	0.68<br>(0.56; 0.81)	0.62<br>(0.51; 0.72)	0.64<br>(0.56; 0.71)	0.58<br>(0.52; 0.64)	0.56<br>(0.52; 0.60)
% Change Mean A-B between Cx1 and Cx2	0.66<br>(0.18; 1.00)	0.78<br>(0.48; 1.00)	0.78<br>(0.58; 0.99)	0.74<br>(0.56; 0.92)	0.73<br>(0.58; 0.89)	0.70<br>(0.57; 0.82)	0.62<br>(0.52; 0.72)	0.64<br>(0.56; 0.71)	0.58<br>(0.52; 0.64)	0.56<br>(0.52; 0.60)
% Change Max A-B between Cx1 and Cx2	0.68<br>(0.20; 1.00)	0.80<br>(0.50; 1.00)	0.79<br>(0.59; 0.99)	0.74<br>(0.56; 0.92)	0.74<br>(0.58; 0.90)	0.69<br>(0.57; 0.82)	0.62<br>(0.51; 0.72)	0.63<br>(0.56; 0.71)	0.57<br>(0.51; 0.63)	0.55<br>(0.51; 0.59)
% Change Min A-C between Cx1 and Cx2	0.69<br>(0.36; 1.00)	0.78<br>(0.57; 1.00)	0.76<br>(0.57; 0.94)	0.71<br>(0.54; 0.88)	0.72<br>(0.57; 0.87)	0.68<br>(0.56; 0.81)	0.64<br>(0.54; 0.75)	0.63<br>(0.55; 0.70)	0.57<br>(0.51; 0.63)	0.54<br>(0.50; 0.58)
% Change Mean A-C between Cx1 and Cx2	0.78<br>(0.43; 0.97)	0.84<br>(0.70; 0.98)	0.79<br>(0.63; 0.96)	0.73<br>(0.57; 0.90)	0.74<br>(0.59; 0.88)	0.69<br>(0.57; 0.81)	0.65<br>(0.54; 0.75)	0.63<br>(0.56; 0.70)	0.57<br>(0.51; 0.63)	0.54<br>(0.50; 0.58)

% Change Max A-C between Cx1 and Cx2	0.81 (0.65; 0.97)	0.86 (0.75; 0.97)	0.80 (0.64; 0.96)	0.74 (0.58; 0.90)	0.74 (0.59; 0.89)	0.69 (0.56; 0.81)	0.64 (0.54; 0.74)	0.62 (0.55; 0.70)	0.56 (0.50; 0.62)	0.53 (0.49; 0.58)

sPTB=spontaneous preterm birth; GW=gestational week

*The endocervical length (distance A-B) was measured as a straight line from the external to the internal cervical os. If the isthmus was present, three distances were measured: the endocervical length (distance A-B), the isthmus length (distance B-C) and the distance A to C
