# Supplementary material for: Second‐trimester transvaginal ultrasound measurement of cervical length for prediction of preterm birth: a blinded prospective multicentre diagnostic accuracy study
Source: BJOG. 2020 Oct 19;128(2):195–206. doi: 10.1111/1471-0528.16519 (PMC7821210; doi:10.1111/1471-0528.16519)
Supplement: Supplementary file 6 — Table S4. Area under the receiver operating characteristic curve for different cervical length measurements with regards to preterm birth at <33 weeks of gestation excluding late miscarriage at 18+0–20+6 weeks of gestation (primary outcome), and with regards to preterm birth including late miscarriage at 18+0–20+6 weeks of gestation, for the three study populations with cervical measurements at 18+0–20+6 weeks of gestation (C×1), at 21+0–23+6 weeks of gestation (C×2), and both at C×1 and C×2 (C×1 C×2). [file BJO-128-195-s006.pdf]

Table S4. Area under the Receiver Operating Characteristic curve for different cervical length measurements with regard to preterm birth <33 weeks excluding late miscarriage at 18+0 to 21+6 weeks (primary outcome) and with regard to preterm birth including late miscarriage at 18+0 to 21+6 weeks for the three study populations with cervical measurements at 18+0 to 20+6 weeks (Cx1), at 21+0 to 23+6 weeks (Cx2), and both at Cx1 and CX 2 (Cx1Cx2)

	**Area under the Receiver Operating Characteristic curve (95% confidence interval)**										
**Cervical measurement***	**Primary outcome PTB <33 GW†**	**PTB <28 GW**	**PTB <29 GW**	**PTB <30 GW**	**PTB <31 GW**	**PTB <32 GW**	**PTB <33 GW**	**PTB <34 GW**	**PTB <35 GW**	**PTB <36 GW**	**PTB <37 GW**
**Cx1 (n=11 072)**	**n=109**	**n=37**	**n=44**	**n=59**	**n=70**	**n=87**	**n=117**	**n=158**	**n=232**	**n=338**	**n=585**
Min A-B (Cx1)	0.57 (0.51; 0.63)	0.73 (0.65; 0.82)	0.73 (0.65; 0.81)	0.68 (0.60; 0.76)	0.67 (0.59; 0.74)	0.63 (0.56; 0.69)	0.59 (0.53; 0.65)	0.59 (0.54; 0.63)	0.57 (0.54; 0.61)	0.58 (0.55; 0.61)	0.57 (0.55; 0.60)
Mean A-B (Cx1)	0.58 (0.52; 0.64)	0.73 (0.65; 0.81)	0.73 (0.65; 0.81)	0.68 (0.60; 0.76)	0.66 (0.59; 0.74)	0.63 (0.56; 0.69)	0.59 (0.54; 0.65)	0.59 (0.54; 0.64)	0.58 (0.54; 0.61)	0.58 (0.55; 0.61)	0.57 (0.55; 0.60)
Max A-B (Cx1)	0.57 (0.51; 0.63)	0.72 (0.65; 0.80)	0.72 (0.64; 0.80)	0.66 (0.59; 0.74)	0.65 (0.58; 0.72)	0.62 (0.55; 0.69)	0.59 (0.53; 0.65)	0.59 (0.54; 0.63)	0.57 (0.53; 0.61)	0.58 (0.55; 0.61)	0.57 (0.55; 0.60)
Min A-C (Cx1)	0.60 (0.54; 0.66)	0.74 (0.65; 0.82)	0.74 (0.66; 0.82)	0.69 (0.61; 0.77)	0.68 (0.61; 0.75)	0.65 (0.58; 0.72)	0.62 (0.56; 0.68)	0.60 (0.55; 0.65)	0.59 (0.55; 0.63)	0.59 (0.55; 0.62)	0.58 (0.56; 0.61)
Mean A-C (Cx1)	0.60 (0.54; 0.66)	0.74 (0.65; 0.82)	0.74 (0.66; 0.82)	0.69 (0.61; 0.77)	0.68 (0.61; 0.76)	0.65 (0.59; 0.72)	0.62 (0.56; 0.68)	0.61 (0.56; 0.66)	0.59 (0.56; 0.63)	0.59 (0.55; 0.62)	0.58 (0.55; 0.60)
Max A-C (Cx1)	0.60 (0.54; 0.66)	0.74 (0.65; 0.82)	0.74 (0.66; 0.82)	0.68 (0.60; 0.76)	0.68 (0.61; 0.75)	0.65 (0.58; 0.72)	0.62 (0.56; 0.68)	0.61 (0.56; 0.66)	0.59 (0.55; 0.63)	0.59 (0.55; 0.62)	0.58 (0.55; 0.60)
Min A-B+B-C (Cx1)	0.60 (0.54; 0.66)	0.74 (0.65; 0.83)	0.74 (0.66; 0.82)	0.69 (0.61; 0.77)	0.68 (0.61; 0.76)	0.65 (0.58; 0.72)	0.62 (0.56; 0.68)	0.60 (0.55; 0.65)	0.59 (0.55; 0.63)	0.58 (0.55; 0.62)	0.58 (0.55; 0.60)
Mean A-B+B-C (Cx1)	0.60 (0.54; 0.66)	0.73 (0.65; 0.82)	0.74 (0.66; 0.82)	0.68 (0.61; 0.76)	0.68 (0.61; 0.75)	0.65 (0.58; 0.72)	0.62 (0.56; 0.68)	0.61 (0.56; 0.66)	0.59 (0.55; 0.63)	0.58 (0.55; 0.62)	0.58 (0.55; 0.60)
Max A-B+B-C (Cx1)	0.60 (0.54; 0.66)	0.72 (0.62; 0.81)	0.72 (0.64; 0.80)	0.67 (0.59; 0.75)	0.67 (0.60; 0.74)	0.64 (0.58; 0.71)	0.62 (0.56; 0.67)	0.60 (0.55; 0.65)	0.59 (0.55; 0.62)	0.58 (0.55; 0.61)	0.57 (0.55; 0.60)
**Cx2 (n = 6288)**	**n=53**	**n=10**	**n=14**	**n=22**	**n=29**	**n=39**	**n=53**	**n=74**	**n=119**	**n=178**	**n=321**
Min A-B (Cx2)	0.59 (0.50; 0.68)	0.57 (0.32; 0.82)	0.69 (0.49; 0.89)	0.62 (0.44; 0.79)	0.64 (0.50; 0.78)	0.61 (0.50; 0.72)	0.59 (0.50; 0.68)	0.61 (0.54; 0.68)	0.63 (0.57; 0.68)	0.62 (0.57; 0.66)	0.60 (0.57; 0.64)
Mean A-B (Cx2)	0.59 (0.51; 0.68)	0.57 (0.32; 0.82)	0.69 (0.48; 0.89)	0.62 (0.44; 0.79)	0.64 (0.50; 0.78)	0.61 (0.50; 0.72)	0.59 (0.51; 0.68)	0.62 (0.55; 0.69)	0.63 (0.57; 0.68)	0.61 (0.57; 0.66)	0.60 (0.57; 0.63)

Max A-B (Cx2)	0.59 (0.50; 0.68)	0.55 (0.31; 0.79)	0.67 (0.47; 0.87)	0.61 (0.44; 0.78)	0.63 (0.49; 0.77)	0.61 (0.50; 0.71)	0.59 (0.50; 0.68)	0.62 (0.55; 0.69)	0.62 (0.57; 0.68)	0.61 (0.57; 0.66)	0.60 (0.56; 0.63)
Min A-C (Cx2)	0.59 (0.50; 0.68)	0.50 (0.24; 0.76)	0.64 (0.42; 0.86)	0.59 (0.42; 0.77)	0.62 (0.47; 0.76)	0.60 (0.49; 0.71)	0.59 (0.50; 0.68)	0.62 (0.55; 0.69)	0.63 (0.57; 0.68)	0.62 (0.57; 0.66)	0.61 (0.57; 0.64)
Mean A-C (Cx2)	0.59 (0.50; 0.68)	0.51 (0.25; 0.77)	0.63 (0.41; 0.77)	0.59 (0.42; 0.68)	0.62 (0.47; 0.76)	0.60 (0.49; 0.74)	0.59 (0.50; 0.68)	0.62 (0.55; 0.69)	0.63 (0.58; 0.69)	0.62 (0.57; 0.66)	0.60 (0.57; 0.64)
Max A-C (Cx2)	0.59 (0.50; 0.68)	0.52 (0.27; 0.78)	0.62 (0.40; 0.84)	0.59 (0.41; 0.76)	0.61 (0.47; 0.76)	0.60 (0.49; 0.71)	0.59 (0.50; 0.68)	0.62 (0.55; 0.69)	0.63 (0.57; 0.68)	0.61 (0.57; 0.66)	0.60 (0.57; 0.63)
Min A-B+B-C (Cx2)	0.59 (0.50; 0.68)	0.50 (0.24; 0.76)	0.64 (0.42; 0.86)	0.59 (0.42; 0.77)	0.61 (0.47; 0.76)	0.60 (0.49; 0.71)	0.59 (0.50; 0.68)	0.62 (0.55; 0.69)	0.63 (0.57; 0.68)	0.62 (0.57; 0.66)	0.61 (0.57; 0.64)
Mean A-B+B-C (Cx2)	0.59 (0.50; 0.68)	0.50 (0.24;0.77)	0.63 (0.42; 0.85)	0.59 (0.42; 0.77)	0.62 (0.47; 0.76)	0.60 (0.49; 0.72)	0.59 (0.50; 0.68)	0.62 (0.55; 0.69)	0.63 (0.57; 0.69)	0.62 (0.57; 0.66)	0.60 (0.57; 0.64)
Max A-B+B-C (Cx2)	0.59 (0.50; 0.68)	0.52 (0.27; 0.77)	0.62 (0.41; 0.84)	0.59 (0.42; 0.76)	0.61 (0.47; 0.76)	0.60 (0.49; 0.71)	0.59 (0.50; 0.68)	0.62 (0.55; 0.69)	0.63 (0.57; 0.68)	0.61 (0.57; 0.66)	0.60 (0.57; 0.63)
**Cx1Cx2 (n=6179)**	**n=52**	**n=9**	**n=13**	**n=21**	**n=28**	**n=38**	**n=52**	**n=71**	**n=115**	**n=171**	**n=313**
Change Min A-B in mm between Cx1 and Cx2	0.60 (0.52; 0.69)	0.63 (0.42; 0.85)	0.49 (0.29; 0.68)	0.51 (0.36; 0.67)	0.55 (0.42; 0.69)	0.58 (0.47; 0.68)	0.60 (0.52; 0.69)	0.58 (0.51; 0.66)	0.59 (0.54; 0.64)	0.56 (0.51; 0.60)	0.55 (0.52; 0.59)
Change Mean A-B in mm between Cx1 and Cx2	0.61 (0.52; 0.70)	0.62 (0.37; 0.86)	0.50 (0.30; 0.71)	0.53 (0.37; 0.69)	0.56 (0.43; 0.70)	0.58 (0.47; 0.69)	0.61 (0.52; 0.70)	0.59 (0.51; 0.66)	0.59 (0.53; 0.65)	0.55 (0.51; 0.60)	0.55 (0.51; 0.58)
Change Max A-B in mm between Cx1 and Cx2	0.60 (0.51; 0.69)	0.62 (0.37; 0.87)	0.51 (0.30; 0.72)	0.54 (0.38; 0.70)	0.56 (0.43; 0.70)	0.58 (0.47; 0.68)	0.60 (0.51; 0.69)	0.58 (0.51; 0.65)	0.59 (0.53; 0.64)	0.55 (0.50; 0.60)	0.54 (0.51; 0.57)
Change Min A-C in mm between Cx1 and Cx2	0.56 (0.48; 0.65)	0.67 (0.46; 0.87)	0.56 (0.38; 0.74)	0.53 (0.38; 0.67)	0.53 (0.40; 0.66)	0.55 (0.44; 0.65)	0.56 (0.48; 0.65)	0.56 (0.49; 0.63)	0.56 (0.51; 0.62)	0.53 (0.49; 0.58)	0.53 (0.49; 0.56)
Change Mean A-C in mm between Cx1 and Cx2	0.57 (0.48;0.65)	0.64 (0.42;0.87)	0.54 (0.35;0.73)	0.55 (0.41;0.70)	0.54 (0.42; 0.67)	0.56 (0.45; 0.66)	0.57 (0.48; 0.65)	0.57 (0.49; 0.64)	0.56 (0.51; 0.62)	0.53 (0.49; 0.58)	0.52 (0.49; 0.56)
Change Max A-C in mm between Cx1 and Cx2	0.57 (0.48; 0.65)	0.63 (0.39; 0.87)	0.53 (0.33; 0.72)	0.57 (0.42; 0.72)	0.56 (0.43; 0.68)	0.56 (0.46; 0.66)	0.57 (0.48; 0.65)	0.56 (0.49; 0.64)	0.56 (0.51; 0.62)	0.53 (0.49; 0.58)	0.52 (0.49; 0.55)
Change Min A-B+B-C in mm between Cx1 and Cx2	0.57 (0.49; 0.65)	0.66 (0.45; 0.86)	0.55 (0.37; 0.73)	0.56 (0.41; 0.70)	0.55 (0.42; 0.67)	0.56 (0.46; 0.66)	0.57 (0.49; 0.65)	0.57 (0.50; 0.64)	0.57 (0.51; 0.62)	0.54 (0.49; 0.58)	0.53 (0.50; 0.56)
Change Mean A-B+B-C in mm between Cx1 and Cx2	0.57 (0.49; 0.66)	0.62 (0.38; 0.86)	0.53 (0.33; 072)	0.58 (0.43; 0.73)	0.56 (0.43; 0.69)	0.57 (0.46; 0.57)	0.57 (0.49; 0.66)	0.57 (0.50; 0.64)	0.57 (0.51; 0.62)	0.53 (0.49; 0.58)	0.52 (0.49; 0.55)
Change Max A-B+B-C in mm between Cx1 and Cx2	0.57 (0.48; 0.65)	0.61 (0.35; 0.87)	0.52 (0.32; 0.72)	0.59 (0.44; 0.74)	0.56 (0.43; 0.70)	0.56 (0.46; 0.67)	0.57 (0.48; 0.65)	0.56 (0.49; 0.63)	0.56 (0.51; 0.61)	0.53 (0.48; 0.57)	0.52 (0.48; 0.55)
% Change Min A-B between Cx1 and Cx2	0.61 (0.52; 0.70)	0.63 (0.40; 0.86)	0.50 (0.29; 0.71)	0.53 (0.37; 0.70)	0.57 (0.43; 0.71)	0.58 (0.47; 0.69)	0.61 (0.52; 0.70)	0.59 (0.52; 0.66)	0.60 (0.54; 0.65)	0.56 (0.51; 0.61)	0.56 (0.52; 0.59)

% Change Mean A-B between Cx1 and Cx2	0.61 (0.52; 0.70)	0.61 (0.36; .86)	0.52 (0.30; 0.74)	0.55 (0.38; 0.72)	0.58 (0.44; 0.72)	0.59 (0.48; 0.70)	0.61 (0.52; 0.70)	0.59 (0.52; 0.67)	0.60 (0.54; 0.66)	0.56 (0.51; 0.61)	0.55 (0.52; 0.58)
% Change Max A-B between Cx1 and Cx2	0.61 (0.52; 0.70)	0.61 (0.35; 0.87)	0.53 (0.31; 0.75)	0.56 (0.40; 0.73)	0.58 (0.44; 0.72)	0.59 (0.48; 0.70)	0.61 (0.52; 0.70)	0.59 (0.51; 0.66)	0.59 (0.54; 0.65)	0.55 (0.51; 0.60)	0.54 (0.51; 0.58)
% Change Min A-C between Cx1 and Cx2	0.57 (0.48; 0.66)	0.66 (0.44; 0.87)	0.47 (0.26; 0.67)	0.55 (0.39; 0.71)	0.55 (0.41; 0.69)	0.56 (0.45; 0.67)	0.57 (0.48; 0.66)	0.57 (0.50; 0.65)	0.57 (0.52; 0.63)	0.54 (0.50; 0.59)	0.53 (0.50; 0.57)
% Change Mean A-C between Cx1 and Cx2	0.58 (0.49; 0.67)	0.63 (0.40; 0.87)	0.52 (0.31; 0.72)	0.57 (0.42; 0.73)	0.57 (0.43; 0.70)	0.57 (0.46; 0.68)	0.58 (0.49; 0.67)	0.58 (0.50; 0.65)	0.58 (0.52; 0.63)	0.54 (0.50; 0.59)	0.53 (0.50; 0.56)
% Change Max A-C between Cx1 and Cx2	0.58 (0.49; 0.67)	0.63 (0.38; 0.88)	0.50 (0.29; 0.71)	0.59 (0.43; 0.75)	0.58 (0.44; 0.71)	0.57 (0.46; 0.68)	0.58 (0.49; 0.67)	0.57 (0.50; 0.65)	0.57 (0.52; 0.63)	0.54 (0.49; 0.59)	0.52 (0.49; 0.56)

PTB=preterm birth; GW=gestational week

* The endocervical length (distance A-B) was measured as a straight line from the external to the internal cervical os. If the isthmus was present, three distances were measured: the endocervical length (distance A-B), the isthmus length (distance B-C) and the distance A to C

† late miscarriage at 18+0 to 21+6 weeks excluded
