# Supplementary material for: Second‐trimester transvaginal ultrasound measurement of cervical length for prediction of preterm birth: a blinded prospective multicentre diagnostic accuracy study
Source: BJOG. 2020 Oct 19;128(2):195–206. doi: 10.1111/1471-0528.16519 (PMC7821210; doi:10.1111/1471-0528.16519)
Supplement: Supplementary file 7 — Table S5. Area under the receiver operating characteristic curve for different cervical length measurements at 18+0–20+6 weeks of gestation (C×1, n = 11 072) for prediction of preterm birth or spontaneous preterm birth at <32, <33 and <34 weeks of gestation in women with, versus without, isthmus. [file BJO-128-195-s007.pdf]

Table S5. Area under the Receiver Operating Characteristic curve for different cervical length measurements at 18+0 to 20+6 GW (Cx1, n=11072) for prediction of preterm birth or spontaneous preterm birth <32, <33 and <34 gestational weeks in women with versus without isthmus

	**Area under the Receiver Operating Characteristic curve (95% confidence interval)**							
	**Birth <33+0 GW* (primary outcome)**		**Spontaneous birth <32+0 GW†**		**Spontaneous birth <33+0 GW†**		**Spontaneous birth <34+0 GW†**	
**Cervical length measurement‡**	**Isthmus present (n=19§)**	**Isthmus absent (n=90§)**	**Isthmus present (n=9§)**	**Isthmus absent (n=37§)**	**Isthmus present (n=12§)**	**Isthmus absent (n=51§)**	**Isthmus present (n=20§)**	**Isthmus absent (n=74§)**
Min A-B	0.59 (0.42; 0.78)	0.74 (0.67; 0.82)	0.51 (0.24; 0.78)	0.76 (0.67; 0.85)	0.57 (0.35; 0.79)	0.74 (0.67; 0.82)	0.51 (0.36; 0.66)	0.70 (0.63; 0.76)
Mean A-B	0.60 (0.44; 0.76)	0.62 (0.56; 0.68)	0.51 (0.26; 0.77)	0.76 (0.67; 0.85)	0.59 (0.38; 0.79)	0.75 (0.67; 0.82)	0.49 (0.35; 0.64)	0.70 (0.64; 0.76)
Max A-B	0.61 (0.46; 0.76)	0.61 (0.55; 0.68)	0.55 (0.32; 0.79)	0.76 (0.67; 0.84)	0.61 (0.42; 0.80)	0.74 (0.67; 0.81)	0.52 (0.39; 0.66)	0.70 (0.64; 0.76)
Min A-C	0.57 (0.42; 0.71)	0.74 (0.67; 0.82)	0.52 (0.27; 0.76)	0.76 (0.67; 0.85)	0.56 (0.35; 0.77)	0.74 (0.67; 0.82)	0.51 (0.34; 0.67)	0.70 (0.63; 0.76)
Mean A-C	0.56 (0.42; 0.71)	0.62 (0.56; 0.68)	0.50 (0.26; 0.75)	0.76 (0.67; 0.85)	0.57 (0.36; 0.78)	0.75 (0.67; 0.82)	0.51 (0.34; 0.67)	0.70 (0.64; 0.76)
Max A-C	0.56 (0.41; 0.71)	0.61 (0.55; 0.68)	0.49 (0.24; 0.74)	0.76 0.67; 0.84)	0.57 (0.36; 0.78)	0.74 (0.67; 0.81)	0.51 (0.34; 0.68)	0.70 (0.64; 0.76)
Min A-B + B-C	0.60 (0.45;0.75)	0.74 (0.67; 0.82)	0.49 (0.24; 0.74)	0.76 (0.67; 0.85)	0.58 (0.37; 0.79)	0.74 (0.67; 0.82)	0.51 (0.35; 0.68)	0.70 (0.63; 0.76)
Mean A-B+ B-C	0.60 (0.44; 0.75)	0.62 (0.56; 0.68)	0.48 (0.23; 0.73)	0.76 (0.67; 0.85)	0.58 (0.37; 0.80)	0.75 (0.67; 0.82)	0.52 (0.36; 0.69)	0.70 (0.64; 0.76)
Max A-B+ B-C	0.60 (0.45; 0.75)	0.61 (0.55; 0.68)	0.56 (0.33; 0.79)	0.76 (0.67; 0.84)	0.60 (0.40; 0.80)	0.74 (0.67; 0.81)	0.55 (0.39; 0.71)	0.70 (0.64; 0.76)

GW= gestational weeks

*does not include late miscarriage

†includes late miscarriage

‡The endocervical length (distance A-B) was measured as a straight line from the external to the internal cervical os. If the isthmus was present, three distances were measured: the endocervical length (distance A-B), the isthmus length (distance B-C) and the distance A to C

§ number of preterm births
