# Supplementary material for: Second‐trimester transvaginal ultrasound measurement of cervical length for prediction of preterm birth: a blinded prospective multicentre diagnostic accuracy study
Source: BJOG. 2020 Oct 19;128(2):195–206. doi: 10.1111/1471-0528.16519 (PMC7821210; doi:10.1111/1471-0528.16519)
Supplement: Supplementary file 8 — Table S6. Shortest endocervical length (distance A–B) at 18+0–20+6 weeks of gestation (C×1) and at 21+0–23+6 weeks of gestation (C×2). [file BJO-128-195-s008.pdf]

Table S6. Shortest endocervical length (distance A-B) at 18+0 to 20+6 weeks (Cx1) and at 21+0 to 23+6 weeks (Cx2)

Cervical length	Cx1 n=11 072		Cx2 n=6288	
	n	%	n	%
≤10 mm	7	0.06	6	0.10
≤15 mm	15	0.14	14	0.22
10-20 mm	61	0.55	65	1.03
≤20 mm	67	0.61	71	1.13
≤25 mm	441	3.98	274	4.36
≤30 mm	2175	19.6	1166	18.6
≤35 mm	5419	48.9	2925	46.5

1st, 5th and 10th percentiles at Cx1: 22 mm, 26 mm, 28 mm

1st, 5th and 10th percentiles at Cx2: 20 mm, 26 mm, 28 mm
