# Supplementary material for: Second‐trimester transvaginal ultrasound measurement of cervical length for prediction of preterm birth: a blinded prospective multicentre diagnostic accuracy study
Source: BJOG. 2020 Oct 19;128(2):195–206. doi: 10.1111/1471-0528.16519 (PMC7821210; doi:10.1111/1471-0528.16519)
Supplement: Supplementary file 9 — Table S7. Area under the receiver operating characteristic curve for different cervical length measurements with regards to preterm birth at <33 weeks of gestation excluding late miscarriage at 18+0–20+6 weeks of gestation (primary outcome), and with regards to spontaneous preterm birth including late miscarriage at 18+0–20+6 weeks of gestation, for the study population with two cervical measurements at 18+0–20+6 weeks of gestation and at 21+0–23+6 weeks of gestation, with at least 14 days between the two measurements (C×1 C×2). [file BJO-128-195-s009.pdf]

Table S7. Area under the Receiver Operating Characteristic curve for different cervical length measurements with regard to preterm birth <33 weeks excluding late miscarriage at 18+0 to 21+6 weeks (primary outcome) and with regard to spontaneous preterm birth including late miscarriage at 18+0 to 21+6 weeks for the study population with two cervical measurements: at 18+0 to 20+6 weeks and at 21+0 to 23+6 weeks with at least 14 days between the two measurements (Cx1Cx2)

	Area under the Receiver Operating Characteristic curve (95% confidence interval)										
Cervical measurement*	Primary outcome PTB <33 GW† n=52	sPTB <28 GW n=3	sPTB <29 GW n=5	sPTB <30 GW n=10	sPTB <31 GW n=15	sPTB 32 GW n=18	sPTB <33 GW n=26	sPTB <34 GW n=40	sPTB <35 GW n=67	sPTB <36 GW n=109	sPTB <37 GW n=220
**Cx1Cx2 (n = 6179)**											
Min A-B (Cx1)	0.51 (0.42; 0.60)	0.97 (0.94; 1.00)	0.97 (0.95; 1.00)	0.78 (0.58; 0.97)	0.73 (0.56; 0.90)	0.67 (0.51; 0.83)	0.65 (0.53; 0.76)	0.66 (0.57; 0.75)	0.62 (0.56; 0.69)	0.62 (0.56; 0.67)	0.59 (0.55; 0.62)
Min A-C (Cx1)	0.55 (0.46; 0.64)	0.88 (0.77; 1.00)	0.92 (0.85; 1.00)	0.79 (0.64; 0.95)	0.76 (0.61; 0.90)	0.67 (0.51; 0.83)	0.66 (0.54; 0.78)	0.64 (0.54; 0.74)	0.62 (0.54; 0.69)	0.61 (0.55; 0.66)	0.59 (0.56; 0.63)
Min A-B+B-C (Cx1)	0.55 (0.46; 0.64)	0.91 (0.77; 1.00)	0.94 (0.86; 1.00)	0.80 (0.64; 0.96)	0.76 (0.61; 0.91)	0.68 (0.51; 0.84)	0.66 (0.54; 0.79)	0.64 (0.53; 0.74)	0.61 (0.54; 0.68)	0.61 (0.55; 0.66)	0.59 (0.55; 0.63)
Min A-B (Cx2)	0.59 (0.50; 0.68)	0.96 (0.92; 1.00)	0.98 (0.95; 1.00)	0.86 (0.69; 1.00)	0.85 (0.72; 0.99)	0.81 (0.68; 0.93)	0.76 (0.65; 0.87)	0.71 (0.63; 0.80)	0.71 (0.64; 0.77)	0.67 (0.62; 0.72)	0.63 (0.59; 0.67)
Min A-C (Cx2)	0.58 (0.49; 0.68)	0.96 (0.93; 1.00)	0.98 (0.95; 1.00)	0.87 (0.71; 1.00)	0.86 (0.73; 0.99)	0.82 (0.71; 0.94)	0.78 (0.68; 0.88)	0.73 (0.65; 0.82)	0.71 (0.65; 0.77)	0.67 (0.61; 0.72)	0.63 (0.60; 0.67)
Min A-B+B-C (Cx2)	0.58 (0.49; 0.68)	0.96 (0.93; 1.00)	0.98 (0.95; 1.00)	0.87 (0.71; 1.00)	0.86 (0.74; 0.99)	0.82 (0.71; 0.94)	0.78 (0.68; 0.88)	0.74 (0.65; 0.82)	0.71 (0.65; 0.77)	0.67 (0.61; 0.72)	0.64 (0.60; 0.67)
Change Min A-B in mm between Cx1 and Cx2	0.60 (0.52; 0.69)	0.54 (0.19; 0.90)	0.68 (0.41; 0.94)	0.71 (0.52; 0.91)	0.69 (0.52; 0.86)	0.69 (0.54; 0.84)	0.67 (0.55; 0.79)	0.60 (0.51; 0.70)	0.63 (0.56; 0.69)	0.58 (0.52; 0.63)	0.56 (0.52; 0.60)
Change Min A-C in mm between Cx1 and Cx2	0.56 (0.48; 0.65)	0.65 (0.35; 0.96)	0.71 (0.51; 0.91)	0.70 (0.53; 0.87)	0.67 (0.51; 0.82)	0.68 (0.54; 0.83)	0.65 (0.53; 0.77)	0.62 (0.52; 0.72)	0.61 (0.54; 0.68)	0.56 (0.50; 0.62)	0.54 (0.50; 0.58)
Change Min A-B+B-C in mm between Cx1 and Cx2	0.57 (0.49; 0.65)	0.65 (0.41; 0.89)	0.71 (0.54; 0.87)	0.70 (0.53; 0.86)	0.66 (0.51; 0.82)	0.68 (0.54; 0.82)	0.65 (0.53; 0.76)	0.62 (0.52; 0.71)	0.61 (0.54; 0.68)	0.56 (0.50; 0.62)	0.54 (0.50; 0.58)
% Change Min A-B between Cx1 and Cx2	0.61 (0.52; 0.70)	0.55 (0.10; 1.00)	0.72 (0.40; 1.00)	0.74 (0.53; 0.96)	0.71 (0.53; 0.90)	0.71 (0.55; 0.87)	0.68 (0.56; 0.81)	0.62 (0.51; 0.72)	0.64 (0.56; 0.71)	0.58 (0.52; 0.64)	0.56 (0.52; 0.60)
% Change Min A-C between Cx1 and Cx2	0.57 (0.48; 0.66)	0.69 (0.36; 1.00)	0.78 (0.57; 1.00)	0.76 (0.57; 0.94)	0.71 (0.54; 0.88)	0.72 (0.57; 0.87)	0.68 (0.56; 0.81)	0.64 (0.54; 0.75)	0.63 (0.55; 0.70)	0.57 (0.51; 0.63)	0.54 (0.50; 0.58)

GW=gestational weeks; PTB=preterm birth; sPTB=spontaneous preterm birth.

*The endocervical length (distance A-B) was measured as a straight line from the external to the internal cervical os If the isthmus was present, three distances were measured: the endocervical length (distance A-B), the isthmus length (distance B-C) and the distance A to C.

†late miscarriage at 18+0 to 21+6 weeks excluded
