# Supplementary material for: Second‐trimester transvaginal ultrasound measurement of cervical length for prediction of preterm birth: a blinded prospective multicentre diagnostic accuracy study
Source: BJOG. 2020 Oct 19;128(2):195–206. doi: 10.1111/1471-0528.16519 (PMC7821210; doi:10.1111/1471-0528.16519)
Supplement: Supplementary file 10 — Table S8. Discriminative ability in terms of positive and negative likelihood ratios, and positive and negative predictive values, for shortest endocervical length (distance A–B) measured at 18+0–20+6 weeks of gestation (C×1, n = 11 072) with regards to predicting spontaneous preterm birth, including late spontaneous miscarriage. [file BJO-128-195-s010.pdf]

Table S8. Discriminative ability in terms of positive and negative likelihood ratios, positive and negative predictive values for shortest endocervical length (distance A-B) measured at 18+0 to 20+6 gestational weeks (Cx1, n=11072) with regard to predicting spontaneous preterm birth including late spontaneous miscarriage

		Cervical length																
		0-15 mm (n=15; 0.14%)				0-20 mm (n=67; 0.6%)				0-25 mm (n=441; 4.0%)				Best cut-off*				
sPTB	No. sPTB (%)	LR+ [95% CI]	LR; [95% CI]	PPV [95% CI]	NPV [95% CI]	LR+ [95% CI]	LR; [95% CI]	PPV [95% CI]	NPV [95% CI]	LR+ [95% CI]	LR; [95% CI]	PPV [95% CI]	NPV [95% CI]	LR+ [95% CI]	LR; [95% CI]	PPV [95% CI]	NPV [95% CI]	mm
<28 GW	22<br>(0.20%)	125.57<br>[38.05; 414.42]	0.86<br>[0.73; 1.02]	3/15<br>(20.0%)<br>[4.3; 48.1]	11038/11057<br>(99.8%)<br>[99.7; 99.9]	31.89<br>[12.71; 80.02]	0.82<br>[0.68; 1.00]	4/67<br>(6.0%)<br>[1.7; 14.6]	10987/11005<br>(99.8%)<br>[99.7; 99.9]	10.46<br>[6.28; 17.44]	0.61<br>[0.43; 0.87]	9/441<br>(2.0%)<br>[0.9; 3.8]	10618/10631<br>(99.9%)<br>[99.8; 99.9]	2.69<br>[2.20; 3.28]	0.26<br>[0.11; 0.63]	18/3383<br>(0.5%)<br>[0.3; 0.8]	7685/7689<br>(99.9%)<br>[99.9; 100.0]	32<br>(n=3383;<br>30.6%)
<29 GW	24<br>(0.22%)	115.08<br>[34.66; 382.13]	0.88<br>[0.75; 1.02]	3/15<br>(20.0%)<br>(4.3; 48.1)	11036/11057<br>(99.8%)<br>[99.7; 99.9]	37.12<br>[16.38; 84.16]	0.80<br>[0.65; 0.98]	5/67<br>(7.5%)<br>[2.5; 16.6]	10986/11005<br>(99.8%)<br>[99.7; 99.9]	10.68<br>(6.59; 17.30]	0.61<br>(0.43; 0.85]	10/441<br>(2.3%)<br>[1.1; 4.1]	10617/10631<br>(99.9%)<br>[99.8; 99.9]	2.74<br>(2.28; 3.28]	0.24<br>(0.10; 0.59]	20/3383<br>(0.6%)<br>[0.4; 0.9]	7685/7689<br>(99.9%)<br>[99.9; 100.0]	32<br>(n=3383;<br>30.6%)
<30 GW	34<br>(0.31%)	81.16<br>[23.97; 274.79]	0.91<br>[0.82; 1.01]	3/15<br>(20.0%)<br>[4.3; 48.1]	11026/11057<br>(99.7%)<br>[99.6; 99.8]	26.18<br>[11.23; 61.05]	0.86<br>[0.75; 0.99]	5/67<br>(7.5%)<br>[2.5; 16.6]	10976/11005<br>(99.7%)<br>[99.6; 99.8]	9.08<br>[5.71; 14.45]	0.67<br>[0.53; 0.86]	12/441<br>(2.7%)<br>[1.4; 4.7]	10609/10631<br>(99.8%)<br>[99.7; 99.9]	2.42<br>[1.97; 2.96]	0.38<br>[0.22; 0.67]	25/3383<br>(0.7%)<br>[0.5; 1.1]	7680/7689<br>(99.9%)<br>[99.8; 100.0]	32<br>(n=3383;<br>30.6%)
<31 GW	40<br>(0.36%)	68.95<br>[20.22; 235.07]	0.93<br>[0.85; 1.01]	3/15<br>(20.0%)<br>[4.3; 48.1]	11020/11057<br>(99.7%)<br>[99.5; 99.8]	27.13<br>[12.45; 59.12]	0.85<br>[0.75; 0.97]	6/67<br>(9.0%)<br>[3.4; 18.5]	10971/11005<br>(99.7%)<br>[99.6; 99.8]	8.38<br>[5.31; 13.22]	0.70<br>[0.57; 0.87]	13/441<br>(2.9%)<br>[1.6; 5.0]	10604/10631<br>(99.7%)<br>[99.6; 99.8]	2.38<br>[1.97; 2.89]	0.40<br>[0.24; 0.65]	29/3383<br>(0.9%)<br>[0.6; 1.2]	7678/7689<br>(99.9%)<br>[99.7; 99.9]	32<br>(n=3383;<br>30.6%)
<32 GW	46<br>(0.42%)	59.92<br>[17.49; 205.34]	0.94<br>[0.87; 1.01]	3/15<br>(20.0%)<br>[4.3; 48.1]	11014/11057<br>(99.6%)<br>[99.5; 99.7]	23.58<br>[10.73; 51.79]	0.87<br>[0.78; 0.98]	6/67<br>(9.0%)<br>[3.4; 18.5]	10965/11005<br>(99.6%)<br>[99.5; 99.7]	7.86<br>[5.03; 12.28]	0.72<br>[0.60; 0.88]	14/441<br>(3.2%)<br>[1.8; 5.3]	10599/10631<br>(99.7%)<br>[99.6; 99.8]	3.41<br>[2.55; 4.57]	0.59<br>[0.44; 0.78]	23/1638<br>(1.4%)<br>[0.9; 2.1]	9411/9434<br>(99.8%)<br>[99.6; 99.9]	29<br>(n=1638;<br>14.8%)
<33 GW	63<br>(0.57%)	43.69<br>[12.63; 151.07]	0.95<br>[0.90; 1.01]	3/15<br>(20.0%)<br>[4.3; 48.1]	10997/11057<br>(99.5%)<br>[99.3; 99.6]	20.39<br>[9.70; 42.84]	0.89<br>[0.82; 0.98]	7/67<br>(10.4%)<br>[4.3; 20.4]	10949/11005<br>(99.5%)<br>(99.3; 99.6)	7.01<br>[4.62; 10.63]	0.76<br>[0.65; 0.88]	17/441<br>(3.9%)<br>[2.3; 6.1]	10585/10631<br>(99.6%)<br>[99.4; 99.7]	2.93<br>[2.19; 3.91]	0.67<br>[0.54; 0.83]	27/1638<br>(1.6%)<br>[1.1; 2.4]	9398/9434<br>(99.6%)<br>[99.5; 99.7]	29<br>(n=1638;<br>14.8%)
<33 GW†	56 (0.51%†)	16.38<br>[2.17; 123.86]	0.98<br>[0.95; 1.02]	1/13<br>(7.7%)<br>[0.2; 36.0]	10996/11051<br>(99.5%)<br>[99.4; 99.6]	16.38<br>[6.84; 39.25]	0.92<br>[0.84; 0.99]	5/65<br>(7.7%)<br>[2.5; 17.1]	10948/10999<br>(99.5%)<br>[99.4; 99.7]	6.03<br>[3.71; 9.79]	0.80<br>[0.69; 0.92]	13/437<br>(3.0%)<br>[1.6; 5.0]	10584/10627<br>(99.6%)<br>[99.5; 99.7]	1.61<br>[1.34; 1.93]	0.56<br>[0.38; 0.81]	38/4682<br>(0.8%)<br>[0.6; 1.1]	6364/6382<br>(99.7%)<br>[99.6; 99.8]	34<br>(n=4682;<br>42.3%)
<34 GW	94<br>(0.85%)	29.20<br>[8.38; 101.78]	0.97<br>[0.93; 1.01]	3/15<br>(20.0%)<br>[4.3; 48.1]	10966/11057<br>(99.2%)<br>[99.0; 99.3]	13.63<br>[6.40; 29.02]	0.93<br>[0.88; 0.99]	7/67<br>(10.4%)<br>[4.3; 20.4]	10918/11005<br>(99.2%)<br>[99.0; 99.4]	5.55<br>[3.72; 8.28]	0.82<br>[0.74; 0.91]	20/441<br>(4.5%)<br>[2.8; 6.9]	10557/10631<br>(99.3%)<br>[99.1; 99.5]	2.55<br>[1.95; 3.33]	0.73<br>[0.63; 0.86]	35/1638<br>(2.1%)<br>[1.5; 3.0]	9375/9434<br>(99.4%)<br>[99.2; 99.5]	29<br>(n=1638;<br>14.8%)
<35 GW	143<br>(1.29%)	27.79<br>[8.96; 86.24]	0.97<br>[0.95; 1.00]	4/15<br>(26.7%)<br>[7.8; 55.1]	10918/11057<br>(98.7%)<br>[98.5; 98.9]	10.36<br>[5.05; 21.29]	0.95<br>[0.91; 0.99]	8/67<br>(11.9%)<br>[5.3; 22.2]	10870/11005<br>(98.8%)<br>[98.6; 99.0]	4.01<br>[2.70; 5.96]	0.88<br>[0.82; 0.94]	22/441<br>(5.0%)<br>[3.2; 7.5]	10510/10631<br>(98.9%)<br>[98.6; 99.1]	1.59<br>[1.34; 1.89]	0.74<br>[0.63; 0.87]	69/3383<br>(2.0%)<br>[1.6; 2.6]	7615/7689<br>(99.0%)<br>[98.8; 99.2]	32<br>(n=3383;<br>30.6%)
<36 GW	226<br>(2.04%)	17.45<br>[5.60; 54.39]	0.98<br>[0.97; 1.00]	4/15<br>(26.7%)<br>[7.8; 55.1]	10835/11057<br>(98.0%)<br>[97.7; 98.3]	7.45<br>[3.74; 14.84]	0.97<br>[0.94; 0.99]	9/67<br>(13.4%)<br>[6.3; 24.0]	10788/11005<br>(98.0%)<br>[97.8; 98.3]	3.50<br>[2.48; 4.95]	0.90<br>[0.86; 0.95]	30/441<br>(6.8%)<br>[4.6; 9.6]	10435/10631<br>(98.2%)<br>[97.9; 98.4]	1.45<br>[1.28; 1.65]	0.75<br>[0.65; 0.86]	118/4013<br>(2.9%)<br>[2.4; 3.5]	6951/7059<br>(98.5%)<br>[98.2; 98.7]	33<br>(n=4013;<br>36.2%)
<37 GW	417<br>(3.77%)	12.78<br>[4.39; 37.21]	0.99<br>[0.98; 1.00]	5/15<br>(33.3%)<br>[11.8; 61.6]	10645/11057<br>(96.3%)<br>[95.9; 96.6]	6.15<br>[3.38; 11.18]	0.99<br>[0.98; 1.00]	13/67<br>(19.4%)<br>[10.8; 30.9]	10601/11005<br>(96.3%)<br>[96.0; 96.7]	2.41<br>[1.75; 3.31]	0.94<br>[0.92; 0.97]	38/441<br>(8.6%)<br>[6.2; 11.6]	10252/10631<br>(96.4%)<br>[96.1; 96.8]	1.39<br>[1.26; 1.54]	0.78<br>[0.71; 0.86]	207/4013<br>(5.2%)<br>[4.5; 5.9]	6849/7059<br>(97.0%)<br>[96.6; 97.4]	33<br>(n=4013;<br>36.2%)

GW=gestational weeks. sPTB=spontaneous preterm birth including miscarriage. No=number of. AUC=area under receiver operating characteristic curve. CI=confidence interval. LR+=positive likelihood ratio. LR-=negative likelihood ratio. PPV=positive predictive value. NPV=negative predictive value.

* best cut-off is the cut-off associated with the largest number of correctly classified cases and is calculated using Youden´s index [21]

† late miscarriage excluded, n=11064 as denominator
