# Supplementary material for: Second‐trimester transvaginal ultrasound measurement of cervical length for prediction of preterm birth: a blinded prospective multicentre diagnostic accuracy study
Source: BJOG. 2020 Oct 19;128(2):195–206. doi: 10.1111/1471-0528.16519 (PMC7821210; doi:10.1111/1471-0528.16519)
Supplement: Supplementary file 11 — Table S9. Discriminative ability in terms of positive and negative likelihood ratios, and positive and negative predictive values, for shortest endocervical length (distance A–B) measured at 21+0–23+6 weeks of gestation (C×2, n = 6288) with regards to predicting spontaneous preterm birth. [file BJO-128-195-s011.pdf]

**Table S9.** Discriminative ability in terms of positive and negative likelihood ratios, positive and negative predictive values for shortest endocervical length (distance A-B) measured at 21+0 to 23+6 gestational weeks (Cx2, n=6288) with regard to predicting spontaneous preterm birth

| | | **Cervical length** | | | | | | | | | | | | | | | | |
|---|---|---|---|---|---|---|---|---|---|---|---|---|---|---|---|---|---|---|
| | | **0-15 mm (n=14; 0.22%)** | | | | **0-20 mm (n=71; 1.1%)** | | | | **0-25 mm (n=274; 4.4%)** | | | | **Best cut-off*** | | | | |
| **sPTB** | **No. sPTB (%)** | **LR+ [95% CI]** | **LR; [95% CI]** | **PPV [95% CI]** | **NPV [95% CI]** | **LR+ [95% CI]** | **LR; [95% CI]** | **PPV [95% CI]** | **NPV [95% CI]** | **LR+ [95% CI]** | **LR; [95% CI]** | **PPV [95% CI]** | **NPV [95% CI]** | **LR+ [95% CI]** | **LR; [95% CI]** | **PPV [95% CI]** | **NPV [95% CI]** | **mm** |
| <28 GW | 3 (0.05) | 161.15 [29.74; 873.33] | 0.67 [0.30; 1.49] | 1/14 (7.1%) [0.2; 33.9] | 6272/6274 (100.0%) [99.9; 100.0] | 29.93 [5.94; 150.81] | 0.67 [0.30; 1.50] | 1/71 (1.4%) [0.0; 7.6] | 6215/6217 (100.0%) [99.9; 100.0] | 7.67 [1.54; 38.18] | 0.70 [0.31; 1.55] | 1/274 (0.4%) [0.0; 2.0] | 6012/6014 (100.0%) [99.9; 100.0] | 12.40 [11.40; 13.48] | 0.00 [.; .] | 3/510 (0.6%) [0.1; 1.7] | 5778/5778 (100.0%) [99.9; 100.0] | 27 (n=510; 8.1%) |
| <29 GW | 5 (0.08) | 342.71 [135.51; 866.70] | 0.40 [0.14; 1.17] | 3/14 (21.4%) [4.7; 50.8] | 6272/6274 (100.0%) [99.9; 100.0] | 55.44 [26.09; 117.80] | 0.40 [0.14; 1.18] | 3/71 (4.2%) [0.9; 11.9] | 6215/6217 (100.0%) [99.9; 100.0] | 13.91 [6.74; 28.72] | 0.42 [0.14; 1.22] | 3/274 (1.1%) [0.2; 3.2] | 6012/6014 (100.0%) [99.9; 100.0] | 12.44 [11.44; 13.53] | 0.00 [.; .] | 5/510 (1.0%) [0.3; 2.3] | 5778/5778 (100.0%) 99.9; 100.0] | 27 (n=510; 8.1%) |
| <30 GW | 10 (0.16) | 348.78 [141.77; 858.04] | 0.50 [0.27; 0.93] | 5/14 (35.7%) [12.8; 64.9] | 6269/6274 (99.9%) [99.8; 100.0] | 57.95 [33.07; 101.54] | 0.40 [0.19; 0.86] | 6/71 (8.5%) [3.2; 17.5] | 6213/6217 (99.9%) [99.8; 100.0] | 14.06 [8.36; 23.63] | 0.42 [0.20; 0.89] | 6/274 (2.2%) [0.8; 4.7] | 6010/6014 (99.9%) [99.8; 100.0] | 10.00 [7.26; 13.79] | 0.22 [0.06; 0.75] | 8/510 (1.6%) [0.7; 3.1] | (100.0%) [99.9; 100.09] | 27 (n=510; 8.1%) |
| <31 GW | 15 (0.24) | 313.65 [123.83; 794.46] | 0.60 [0.40; 0.91] | 6/14 (42.9%) [17.7; 71.1] | 6265/6274 (99.9%) [99.7; 99.9] | 53.10 [31.15; 90.52] | 0.47 [0.27; 0.81] | 8/71 (11.3%) [5.0; 21.0] | 6210/6273 (99.0%) [98.7%; 99.2%] | 12.58 [7.72; 20.48] | 0.49 [0.28; 0.84] | 8/274 (2.9%) [1.3; 5.7] | 6007/6014 (99.9%) [99.8; 100.0] | 9.22 [6.72; 12.65] | 0.29 [0.13; 0.67] | 11/510 (2.2%) [1.1; 3.8] | 5774/5778 (99.9%) (99.8; 100.0) | 27 (n=510; 8.1%) |
| <32 GW | 18 (0.29) | 261.25 [100.83; 676.91] | 44.23 [24.97; 78.37] | 6/14 (42.9%) [17.7; 71.1] | 6262/6274 (99.8%) [99.7; 99.9] | 44.23 [24.97; 78.37] | 0.56 [0.37; 0.85] | 8/71 (11.3%) [5.0; 21.0] | 6207/6217 (99.8%) [99.7; 99.9] | 10.48 [6.17; 17.79] | 0.58 [0.38; 0.88] | 8/274 (2.9%) [1.3; 5.7] | 6004/6014 (99.8%) [99.7; 99.9] | 7.68 [5.26; 11.21] | 0.42 [0.24; 0.75] | 11/510 (2.2%) [1.1; 3.8] | 5771/5778 (99.9%) [99.8; 100.0] | 27 (n=510; 8.1%) |
| <33 GW | 26 (0.41) | 180.63 [67.39; 484.17] | 0.77 [0.62; 0.95] | 6/14 (42.9%) [17.7; 71.1] | 6254/6274 (99.7%) [99.5; 99.8] | 34.96 [19.51; 62.66] | 0.66 [0.50; 0.87] | 9/71 (12.7%) [6.0; 22.7] | 6200/6217 (99.7%) [99.6; 99.8] | 9.12 [5.53; 15.05] | 0.64 [0.47; 0.87] | 10/274 (3.6%) [1.8; 6.6] | 5998/6014 (99.7%) [99.6; 99.9] | 6.80 [4.72; 9.80] | 0.50 [0.33; 0.76] | 14/510 (2.7%) [1.5; 4.6] | 5766/5778 (99.8%) 99.6; 99.99 | 27 (n=510; 8.1%) |
| <34 GW | 41 (0.65) | 114.27 [41.50; 314.69] | 0.85 [0.75; 0.97] | 6/41 (14.6%) [5.6%; 29.2%] | 6239/6247 (99.9%) [99.8%; 99.9%] | 24.98 [13.79; 45.24] | 0.76 [0.64; 0.91] | 10/41 (24.4%) [12.4%; 40.3%] | 6186/6247 (99.0%) [98.8%; 99.3%] | 6.98 [4.27; 11.40] | 0.74 [0.61; 0.90] | 12/274 (4.4%) [2.3; 7.5] | 5985/6014 (99.5%) [99.3; 99.7] | 4.93 [3.33; 7.30] | 0.66 [0.52; 0.85] | 16/510 (3.1%) [1.8; 5.0] | 5753/5778 (99.6%) [99.4; 99.7] | 27 (n=510; 8.1%) |
| <35 GW | 69 (1.10) | 90.13 [32.49; 250.06] | 0.90 [0.83; 0.97] | 7/14 (50.0%) [23.0; 77.0] | 6212/6274 (99.0%) [98.7; 99.2] | 20.20 [11.62; 35.11] | 0.82 [0.73; 0.92] | 13/71 (18.3%) [10.1; 29.3] | 6161/6217 (99.1%) [98.8; 99.3] | 5.96 [3.88; 9.16] | 0.79 [0.69; 0.90] | 17/274 (6.2%) [3.7; 9.8] | 5962/6014 (99.1%) [98.9; 99.4] | 1.66 [1.46; 1.90] | 0.43 [0.28; 0.66] | 53/2926 (1.8%) ]1.4; 2.4] | 3346/3362 (99.5%) [99.2; 99.7] | 35 (n=2926; 46.5%) |
| <36 GW | 114 (1.81) | 54.16 [19.31; 151.88] | 0.94 [0.90; 0.98] | 7/14 (50.0%) [23.0; 77.0] | 6167/6274 (98.3%) [97.9; 98.6] | 14.51 [8.46; 24.87] | 0.88 [0.82; 0.94] | 15/71 (21.1%) [12.3; 32.4] | 6118/6217 (98.4%) [98.1; 98.7] | 4.96 [3.38; 7.29] | 0.83 [0.76; 0.91] | 23/274 (8.4%) [5.4; 12.3] | 5923/6014 (98.5%) [98.2; 98.8] | 1.54 [1.37; 1.74] | 0.54 [0.40; 0.72] | 81/2926 (2.8%) [2.2; 3.4] | 3329/3362 (99.0%) [98.6; 99.3] | 35 (n=2926; 46.5%) |
| <37 GW | 225 (3.58) | 26.95 [9.53; 76.18] | 0.97 [0.95; 0.99] | 7/14 (50.0%) [23.0; 77.0] | 056/6274 (96.5%) [96.0; 97.0] | 9.15 [5.45; 15.36] | 0.93 [0.89; 0.96] | 18/71 (25.4%) [15.8; 37.1] | 6010/6217 (96.7%) [96.2; 97.1] | 3.82 [2.73; 5.33] | 0.88 [0.84; 0.93] | 34/274 (12.4%) [8.8; 16.9] | 5823/6014 (96.8%) [96.4; 97.3] | 1.39 [1.26; 1.54] | 0.67 [0.56; 0.79] | 144/2926 (4.9%) [4.2; 5.8] | 3281/3362 (97.6%) [97.0; 98.1] | 35 (n=2926; 46.5%) |

GW=gestational weeks. sPTB=spontaneous preterm birth. No=number of. AUC=area under receiver operating characteristic curve. CI=confidence interval. LR+ = positive likelihood ratio. LR-=negative likelihood ratio. PPV=positive predictive value. NPV=negative predictive value.

* best cut-off is the cut-off associated with the largest number of correctly classified cases and is calculated using Youden´s index [21]
