# Supplementary material for: Second‐trimester transvaginal ultrasound measurement of cervical length for prediction of preterm birth: a blinded prospective multicentre diagnostic accuracy study
Source: BJOG. 2020 Oct 19;128(2):195–206. doi: 10.1111/1471-0528.16519 (PMC7821210; doi:10.1111/1471-0528.16519)
Supplement: Supplementary file 12 — Table S10. Discriminative ability of shortest endocervical length (distance A–B) of 0–30 mm at 18+0–20+6 weeks of gestation (C×1, n = 11 072) with regards to predicting spontaneous preterm birth, including late spontaneous miscarriage. [file BJO-128-195-s012.pdf]

Table S10. Discriminative ability of shortest endocervical length (distance A-B) 0-30 mm measured at 18+0 to 20+6 gestational weeks (Cx1) with regard to predicting spontaneous preterm birth including late spontaneous miscarriage

**Cx1 (n=11072)**									
**Shortest endocervical length 0-30 mm (n=2175; 19.6%)**									
**sPTB**	**No. sPTB**	**AUC (95% CI)**	**Sensitivity [95% CI]**	**Specificity [95% CI]**	**FP/TP; NNS**	**LR+ [95% CI]**	**LR- [95% CI]**	**PPV [95% CI]**	**NPV [95% CI]**
**<28 GW**	22 (0.20%)	0.83 (0.74; 0.92)	14/22 (63.6%) [40.7; 82.8]	8889/11050 (80.4%) [79.7; 81.2]	154; 791	3.25 [2.37; 4.47]	0.45 [0.26; 0.79]	14/2175 (0.6%) [0.4; 1.1]	8889/8897 (99.9%) [99.8; 100.0]
**<29 GW**	24 (0.22%)	0.84 (0.76; 0.92)	16/24 (66.7%) [44.7; 84.4]	8889/11048 (80.5%) [79.7; 81.2]	135; 692	3.41 [2.56; 4.54]	0.41 [0.24; 0.73]	16/2175 (0.7%) [0.4-1.2]	8889/8897 (99.9%) [99.8; 100.0]
**<30 GW**	34 (0.31%)	0.77 (0.68; 0.87)	21/34 (61.8%) [43.6; 77.8]	8884/11038 (80.5%) [79.7; 81.2]	103; 527	3.17 [2.42; 4.13]	0.48 [0.31; 0.73]	21/2175 (1.0%) [0.6; 1.5]	8884/8897 (99.9%) [99.8; 99.9]
**<31 GW**	40 (0.36%)	0.76 (0.67; 0.85)	24/40 (60.0%) [43.3; 75.1]	8881/11032 (80.5%) [79.8; 81.2]	90; 461	3.08 [2.38; 3.97]	0.50 [0.34; 0.73]	24/2175 (1.1%) [0.7; 1.6]	8881/8897 (99.8%) [99.7; 99.9]
**<32 GW**	46 (0.42%)	0.71 (0.62; 0.80)	25/46 (54.3%) [39.0; 69.1]	8876/11026 (80.5%) [79.8; 81.2]	86; 443	2.79 [2.13; 3.64]	0.57 [0.41; 0.78]	25/2175 (1.1%) [0.8; 1.7]	8876/8897 (99.8%) [99.6-; 9.9]
**<33 GW**	63 (0.57%)	0.68 (0.60; 0.76)	29/63 (46.0%) [33.4; 59.1]	8863/11009 (80.5%) [79.8; 81.2]	74; 382	2.36 [1.80; 3.09]	0.67 [0.53; 0.84]	29/2175 (1.3%) [0.9; 1.9]	863/8897 (99.6%) [99.5; 99.7]
**<33 GW†**	56 (0.51%†)	0.66 (0.58; 0.74)	23/56 (41.1%) [28.1; 55.0]	8862/11008 (80.5%) [79.8; 81.2]	93; 481	2.11 [1.54; 2.89]	0.73 [0.59; 0.91]	23/2169 (1.1%) [0.7; 1.6]	8862/8895 (99.6%) [99.5; 99.7]
**<34 GW**	94 (0.85%)	0.65 (0.59; 0.72)	39/94 (41.5%) [31.4; 52.1]	8842/10978 (80.5%) [79.8; 81.3]	55; 284	2.13 [1.67; 2.72]	0.73 [0.61; 0.86]	39/2175 (1.8%) [1.3; 2.4]	8842/8897 (99.4%) [99.2; 99.5]
**<35 GW**	143 (1.29%)	0.62 (0.57; 0.67)	50/143’ (35.0%) [27.2; 43.4]	8804/10929 [80.6%] [79.8; 81.3]	43; 221	1.80 [1.43; 2.26]	0.81 [0.72; 0.91]	50/2175 (2.3%) [1.7; 3.0]	8804/8897 (99.0%) [98.7; 99.2]
**<36 GW**	226 (2.04%)	0.61 (0.57; 0.65)	76/226 (33.6%) [27.5; 40.2]	8747/10846 [80.6%] [79.9; 81.4]	28; 146	1.74 [1.44; 2.10]	0.82 [0.75; 0.90]	76/2175 (3.5%) [2.8; 4.4]	8747/8897 (98.3%) [98.0; 98.6]
**<37 GW**	417 (3.77%)	0.60 (0.57; 0.63)	130/417 (31.2%) [26.8; 35.9]	8610/10655 [80.8%] [80.1; 81.6]	16; 85	1.62 [1.40; 1.88]	0.85 [0.80; 0.91]	130/2175 (6.0%) [5.0; 7.1]	8610/8897 (96.8%) [96.4; 97.1]

GW=gestational weeks. sPTB=spontaneous preterm birth including late miscarriage. No.=number of. AUC=area under receiver operating characteristic curve. CI=confidence interval. FP=false positive. TP=true positive. NNS=number needed to screen, i.e. number of women needed to screen to detect one true positive test result. LR+ = positive likelihood ratio. LR- = negative likelihood ratio. PPV=positive predictive value. NPV=negative predictive value.

† late miscarriage excluded, n=11064 as denominator for Cx1
