# Supplementary material for: Second‐trimester transvaginal ultrasound measurement of cervical length for prediction of preterm birth: a blinded prospective multicentre diagnostic accuracy study
Source: BJOG. 2020 Oct 19;128(2):195–206. doi: 10.1111/1471-0528.16519 (PMC7821210; doi:10.1111/1471-0528.16519)
Supplement: Supplementary file 13 — Table S11. Discriminative ability of shortest endocervical length (distance A–B) of 0–30 mm at 21+0–23+6 weeks of gestation (C×2, n = 6288) with regards to predicting spontaneous preterm birth. [file BJO-128-195-s013.pdf]

Table S11. Discriminative ability of shortest endocervical length (distance A-B) 0-30 mm measured at 21+0 to 23+6 gestational weeks (Cx2) with regard to predicting spontaneous preterm birth

**Cx2 [n=6288]**									
**Shortest endocervical length 0-30 mm (n=1168; 18.6%)**									
**sPTB**	**No. sPTB**	**AUC (95% CI)**	**Sensitivity [95% CI]**	**Specificity [95% CI]**	**FP/TP; NNS**	**LR+ [95% CI]**	**LR- [95% CI]**	**PPV [95% CI]**	**NPV [95% CI]**
**<28 GW**	3 (0.05%)	0.96 (0.92; 1.00)	3/3 (100%) [29.2; 100]	5120/6285 (81.5%) [80.5; 82.4]	388; 2096	5.39 [5.12; 5.68]	NA	3/1168 (0.3%) (0.1; 0.8)	5120/5120 (100.0%) (99.9; 100.0]
**<29 GW**	5 (0.08%)	0.98 (0.95; 1.00)	5/5 (100%) [47.8; 100]	5120/6283 (81.5%) [80.5; 82.4]	233; 1258	5.40 [5.13; 5.69]	NA	5/1168 (0.4%) [0.1; 1.0]	5120/5120 (100.0%) [99.9; 100.0]
**<30 GW**	10 (0.16%)	0.86 (0.69; 1.00)	8/10 (80.0%) [44.7; 97.5]	5118/6278 (81.5%) [80.5; 82.5]	145; 786	4.33 [3.16; 5.93]	0.25 [0.07; 0.85]	8/1168 (0.7%) [0.3; 1.4]	5118/5120 (100.0%) [99.9; 100.0]
**<31 GW**	15 (0.24%)	0.85 (0.72; 0.99)	12/15 (80.0%) [51.9; 95.7]	5117/6273 (81.6%) [80.6; 82.5]	96; 524	4.34 [3.35; 5.62]	0.25 [0.09; 0.67]	12/1168 (1.0%) [0.5; 1.8]	5117/5120 (99.9%) [99.8; 100.0]
**<32 GW**	18 (0.29%)	0.81 (0.68; 0.93)	12/18 (66.7%) [41.0; 86.7]	5114/6270 (81.6%) [80.6; 82.5]	96; 524	3.62 [2.60; 5.03]	0.41 [0.21; 0.79]	12/1168 (1.0%) [0.5; 1.8]	5114/5120 (99.9%) [99.8; 100.0]
**<33 GW**	26 (0.41%)	0.76 (0.65; 0.87)	15/26 (57.7%) [36.9; 76.7]	5109/6262 (81.6%) [80.6; 82.5]	77; 419	3.13 [2.25; 4.37]	0.52 [0.33; 0.81]	15/1168 (1.3%) [0.7; 2.1]	5109/5120 (99.8%) [99.6; 99.9]
**<34 GW**	41 (0.65%)	0.71 (0.63; 0.80)	20/41 (48.8%) [32.9; 64.9]	5099/6247 (81.6%) [80.6; 82.6]	57; 314	2.65 [1.93; 3.65]	0.63 [0.47; 0.85]	20/1168 (1.7%) [1.1; 2.6]	5099/5120 (99.6%) [99.4; 99.8]
**<35 GW**	69 (1.10%)	0.71 (0.65; 0.77)	31/69 (44.9%) [32.9; 57.4]	5082/6219 (81.7%) [80.7; 82.7]	37; 203	2.46 [1.88; 3.21]	0.67 [0.54; 0.83]	31/1168 (2.7%) [1.8; 3.8]	5082/5120 (99.3%) [99.0; 99.5]
**<36 GW**	114 (1.81%)	0.67 (0.61; 0.72)	42/114 (36.8%) [28.0; 46.4]	5048/6174 (81.8%) [80.8; 82.7]	27; 150	2.02 [1.58; 2.58]	0.77 [0.67; 0.89]	42/1168 (3.6%) [2.6; 4.8]	5048/5120 (98.6%) [98.2; 98.9]
**<37 GW**	225 (3.58%)	0.63 (0.59; 0.67)	73/225 (32.4%) [26.4; 39.0]	4968/6063 (81.9%) [81.0; 82.9]	15; 86	1.80 [1.48; 2.19]	0.82 [0.75; 0.90]	73/1168 (6.3%) [4.9; 7.8]	4968/5120 (97.0%) [96.5; 97.5]

GW=gestational weeks. sPTB=spontaneous preterm birth. No.=number of. AUC=area under receiver operating characteristic curve. CI=confidence interval. FP=false positive. TP=true positive. NNS=number needed to screen, i.e. number of women needed to screen to detect one true positive test result. NA, not applicable (100% sensitivity). LR+ = positive likelihood ratio. LR- = negative likelihood ratio. PPV=positive predictive value. NPV=negative predictive value.

There were no late miscarriages in the Cx2 group
